# Correction: Prevalence and factors associated with hepatitis B and C virus infections among female Sex workers in Ethiopia: Results of the national biobehavioral Survey, 2020

**DOI:** 10.1371/journal.pone.0292824

**Published:** 2023-10-09

**Authors:** Birra Bejiga Bedassa, Gemechu Gudeta Ebo, Jemal Ayalew Yimam, Jaleta Bulti Tura, Feyiso Bati Wariso, Sileshi Lulseged, Getachew Tollera Eticha, Tsigereda Kifle Wolde, Saro Abdella Abrahim

There are errors in the Funding section. The correct Funding statement is: The survey was conducted using funding support obtained from the Government of Ethiopia Federal HIV Prevention and Control Office (FHAPCO) budget line ETH-H-HAPCO-1553. The findings and conclusions in this report are those of the authors and do not necessarily represent the official position of the funding organizations. The funders had no role in study design, data collection, analysis, decision to publish, or preparation of the manuscript.
