# Supplementary material for: Prevalence and factors associated with hepatitis B and C virus infections among female Sex workers in Ethiopia: Results of the national biobehavioral Survey, 2020
Source: PLoS One. 2022 Dec 30;17(12):e0269510. doi: 10.1371/journal.pone.0269510 (PMC9803120; doi:10.1371/journal.pone.0269510)
Supplement: Attachment — Submitted filename: PLOS_ONE-D-22-14713_reviewer (reviewed).pdf [file pone.0269510.s001.pdf]

PLOS ONE

Cover letter

A new report examines Hepatitis B and C Virus infections in one of the world's most vulnerable populations. It will have a significant impact on infection control policies and strategies at the local, regional, and global levels.

--Manuscript Draft--

**Manuscript Number:**	PONE-D-22-14713
**Article Type:**	Research Article
**Full Title:**	Cover letter<br>A new report examines Hepatitis B and C Virus infections in one of the world's most vulnerable populations. It will have a significant impact on infection control policies and strategies at the local, regional, and global levels.
**Short Title:**	Prevalence and factors associated with hepatitis B and C virus infections among female Sex workers in Ethiopia
**Corresponding Author:**	Biraa Bejiga, MPH<br>Ethiopian Public Health Institute<br>Addis Ababa, ETHIOPIA
**Keywords:**	hepatitis B; hepatitis C; female sex workers; syphilis; Ethiopia
**Abstract:**	Background: Hepatitis B and C virus infections are endemic diseases in sub-Saharan Africa, the region with the highest prevalence of these infections in the world. Female sex workers are exposed to sexually transmitted infections, including hepatitis B and C, because of their high-risk sexual behavior and limited access to health services. There is no data on national prevalence estimate on hepatitis B and C virus infections among female sex workers in Ethiopia, a critical gap in information this study aimed to fill.<br>Methods: This was a cross-sectional, biobehavioral, national study conducted from December 2019 - April 2020 among 6085 commercial female sex workers aged ≥15 years and residing in sixteen (n=16) regional capital cities and selected towns of Ethiopia. Blood samples were collected for hepatitis B and C virus serological testing from the participants. The data were collected using an open data kits (ODK) software and imported into STATA version16 for analysis. Descriptive statistics (frequencies and proportions) were used to summarize data on the study variables. Bivariate and multivariate logistic regression analyses were conducted to determine the strength of association between independent variables (risk factors) and the outcome (hepatitis B and C virus infection). Adjusted Odd ratio (AOR) was used to determine independent associations, 95% confidence interval to assess precision of the estimates, and a P value ≤ 0.05 was considered statistically significant.<br>Results: The prevalence of hepatitis B and C infections among the 6085 female sex workers was 2.6% [(95% CI (2.2,2.8)] and 0.5% [(95% CI (0.4,0.7)], respectively. Female sex workers who had 61-90 and ≥91 paying clients in the past six months [(AOR=1.66; 95% CI, (0.99, 2.79); P =0.054] and [(AOR=1.66 95% CI, (1.11, 2.49); P=0.013], respectively, age at first sex selling of 20-24 and >25 years [(AOR=1.67; 95% CI, (1.14, 2.44); P =0.009)] and [(AOR=1.56; 95% CI (1.004, 2.43); P =0.048)], respectively, known HIV positive status [(AOR=1.64; 95% CI (1.03, 2.62); P =0.036] were significantly associated with the prevalence of hepatitis B virus infection. Similarly, hepatitis C was significantly associated with, age at first sex ≤15 years and age 16-20 years [(AOR=0.21; 95%CI (0.07,0.61); P =0.005)] and [(AOR=0.18; 95% CI (0.061, 0.53); P =0.002)], respectively, known HIV positive status [(AOR=2.85; 95%CI (1.10,7.37); P =0.031)] and testing positive for syphilis [(AOR=4.38; 95% CI (1.73,11.11); P =0.002)], respectively.<br>Conclusion: This analysis reveals an intermediate prevalence of hepatitis B and a low prevalence of hepatitis C infection among female sex workers in Ethiopia. It also suggests that population groups like female sex workers are highly vulnerable to hepatitis B, hepatitis C, and other sexually transmitted infections. There is a need for strengthening treatment and prevention interventions, including immunization services.
**Order of Authors:**	Biraa Bejiga, MPH(Epidemiology and Biostatistics)
	Gemechu Gudeta Ebo, Masters of Public Health (MPH)

Powered by Editorial Manager® and ProduXion Manager® from Aries Systems Corporation

	Jemal Ayalew Yimam, PHD
	Jaleta Bulti Tur, Masters of Public Health (MPH)
	Feyiso Bati Wariso, Masters of Public Health (MPH)
	Silesh Lulseged
	Getachew Tollera Eticha, Medical Doctor(MPH
	Tsigereda Kifle Wolde, Medical Doctor(MPH
	Saro Abdella Abrahim, Masters of Public Health (MPH)
**Additional Information:**	
**Question**	**Response**
**Financial Disclosure**<br><br>Enter a financial disclosure statement that describes the sources of funding for the work included in this submission. Review the submission guidelines for detailed requirements. View published research articles from *PLOS ONE* for specific examples.<br><br>This statement is required for submission and **will appear in the published article** if the submission is accepted. Please make sure it is accurate.<br><br>**Unfunded studies**<br>Enter: *The author(s) received no specific funding for this work.*<br><br>**Funded studies**<br>Enter a statement with the following details:<br>• Initials of the authors who received each award<br>• Grant numbers awarded to each author<br>• The full name of each funder<br>• URL of each funder website<br>• Did the sponsors or funders play any role in the study design, data collection and analysis, decision to publish, or preparation of the manuscript?<br>• **NO** - Include this sentence at the end of your statement: *The funders had no role in study design, data collection and analysis, decision to publish, or preparation of the manuscript.*<br>• **YES** - Specify the role(s) played.<br><br>* typeset	This project was conducted using the effort of many institutions, organizations, and individuals without whose contributions could not have been possible like, FHAPCO, MOH, CDC, ICAP, PSI and Regional health bureau the U.S. President's Emergency Plan for AIDS Relief (PEPFAR) funds obtained though the U.S Center for Disease Control and Prevention (CDC) under the term of cooperative agreement #U2GGH001226. The findings and conclusions in this report are those of the authors and do not necessarily represent the official position of the funding agency.
**Competing Interests**	The authors have declared that no competing interests exist

Use the instructions below to enter a competing interest statement for this submission. On behalf of all authors, disclose any competing interests that could be perceived to bias this work—acknowledging all financial support and any other relevant financial or non-financial competing interests.<br><br>This statement is required for submission and **will appear in the published article** if the submission is accepted. Please make sure it is accurate and that any funding sources listed in your Funding Information later in the submission form are also declared in your Financial Disclosure statement.<br><br>View published research articles from *PLOS ONE* for specific examples.<br><br>**NO authors have competing interests**<br><br>Enter: *The authors have declared that no competing interests exist.*<br><br>**Authors with competing interests**<br><br>Enter competing interest details beginning with this statement:<br><br>*I have read the journal's policy and the authors of this manuscript have the following competing interests: [insert competing interests here]*<br><br>* typeset	
**Ethics Statement**<br><br>Enter an ethics statement for this submission. This statement is required if the study involved:<br><br>• Human participants<br>• Human specimens or tissue<br>• Vertebrate animals or cephalopods<br>• Vertebrate embryos or tissues<br>• Field research	Ethical approval for the study protocol was obtained from the Scientific and Ethical Research Office (SERO) of the Ethiopian Public Health Institute (EPHI). Potential participants were told about the study purpose and procedures, potential risks, and protections using the local language. Written informed consent was obtained from each survey participant for the interview, blood sample collection, and storage of biospecimens for future testing.

Powered by Editorial Manager® and ProduXion Manager® from Aries Systems Corporation

Write "N/A" if the submission does not require an ethics statement.

General guidance is provided below. Consult the submission guidelines for detailed instructions. **Make sure that all information entered here is included in the Methods section of the manuscript.**

Format for specific study types

Human Subject Research (involving human participants and/or tissue)

- Give the name of the institutional review board or ethics committee that approved the study
- Include the approval number and/or a statement indicating approval of this research
- Indicate the form of consent obtained (written/oral) or the reason that consent was not obtained (e.g. the data were analyzed anonymously)

Animal Research (involving vertebrate animals, embryos or tissues)

- Provide the name of the Institutional Animal Care and Use Committee (IACUC) or other relevant ethics board that reviewed the study protocol, and indicate whether they approved this research or granted a formal waiver of ethical approval
- Include an approval number if one was obtained
- If the study involved *non-human primates*, add *additional details* about animal welfare and steps taken to ameliorate suffering
- If anesthesia, euthanasia, or any kind of animal sacrifice is part of the study, include briefly which substances and/or methods were applied

Field Research

Include the following details if this study involves the collection of plant, animal, or other materials from a natural setting:

- Field permit number
- Name of the institution or relevant body that granted permission

Powered by Editorial Manager® and ProduXion Manager® from Aries Systems Corporation

**Data Availability**<br><br>Authors are required to make all data underlying the findings described fully available, without restriction, and from the time of publication. PLOS allows rare exceptions to address legal and ethical concerns. See the PLOS Data Policy and FAQ for detailed information.<br><br>A Data Availability Statement describing where the data can be found is required at submission. Your answers to this question constitute the Data Availability Statement and **will be published in the article**, if accepted.<br><br>**Important:** Stating 'data available on request from the author' is not sufficient. If your data are only available upon request, select 'No' for the first question and explain your exceptional situation in the text box.<br><br>Do the authors confirm that all data underlying the findings described in their manuscript are fully available without restriction?	Yes - all data are fully available without restriction
**Describe where the data may be found in full sentences. If you are copying our sample text, replace any instances of XXX with the appropriate details.**<br><br>• If the data are **held or will be held in a public repository**, include URLs, accession numbers or DOIs. If this information will only be available after acceptance, indicate this by ticking the box below. For example: *All XXX files are available from the XXX database (accession number(s) XXX, XXX.*).<br>• If the data are all contained **within the manuscript and/or Supporting Information files**, enter the following: *All relevant data are within the manuscript and its Supporting Information files.*<br>• If neither of these applies but you are able to provide **details of access elsewhere**, with or without limitations, please do so. For example:	By following the Ethiopian Public Health Institute's data usage laws and regulations, data-set documentation is available for immediate download and datasets are available upon request for access and depository

Data cannot be shared publicly because of [XXX]. Data are available from the XXX Institutional Data Access / Ethics Committee (contact via XXX) for researchers who meet the criteria for access to confidential data.<br><br>*The data underlying the results presented in the study are available from (include the name of the third party and contact information or URL).*<br>• This text is appropriate if the data are owned by a third party and authors do not have permission to share the data.<br><br>* typeset	
Additional data availability information:	Tick here if your circumstances are not covered by the questions above and you need the journal's help to make your data available.

Manuscript Click here to access/download;Manuscript;Manuscript.docx

Prevalence and factors associated with hepatitis B and C virus infections among female Sex workers in Ethiopia: Results of the national biobehavioral Survey, 2020

Bira Bejiga Bedassa[1*¶], Gemechu Gudeta Ebo[1¶], Jemal Ayalew[1¶], Jaleta Bulti Tura[1&], Feyiso Bati Wariso[1&], Sileshi Lulseged[3&], Getachew Tollera[1&], Tsigereda Kifle[1&], Saro Abdella[1,2¶]

[1]HIV/AIDS disease research team, TB and HIV/AIDS Disease Research Directorate, Ethiopian Public Health Institute, Addis Ababa, Ethiopia

[2]TB disease research team, TB and HIV/AIDS Disease Research Directorate, Ethiopian Public Health Institute, Addis Ababa, Ethiopia

[3]Faculty of Medicine, College of Health Sciences, Addis Ababa University, Ethiopia

[1]Deputy Director General, Ethiopian Public Health Institute, Addis Ababa, Ethiopia

[1] Director General, Ethiopian Public Health Institute, Addis Ababa, Ethiopia

***Corresponding author:** E-mail: birr4allephi@gmail.com

[¶]These authors contributed equally to this work.

[&]This author contributed less but substantially.

ABSTRACT

Background: Hepatitis B and C virus infections are endemic diseases in sub-Saharan Africa, the region with the highest prevalence of these infections in the world. Female sex workers are exposed to sexually transmitted infections, including hepatitis B and C, because of their high-risk sexual behavior and limited access to health services. There is no data on national prevalence estimate on hepatitis B and C virus infections among female sex workers in Ethiopia, a critical gap in information this study aimed to fill.

Methods: This was a cross-sectional, biobehavioral, national study conducted from December 2019 - April 2020 among 6085 commercial female sex workers aged ≥15 years and residing in sixteen (n=16) regional capital cities and selected towns of Ethiopia. Blood samples were collected for hepatitis B and C virus serological testing from the participants. The data were collected using an open data kits (ODK) software and imported into STATA version16 for analysis. Descriptive statistics (frequencies and proportions) were used to summarize data on the study variables. Bivariate and multivariate logistic regression analyses were conducted to determine the strength of association between independent variables (risk factors) and the outcome (hepatitis B and C virus infection). Adjusted Odd ratio (AOR) was used to determine independent associations, 95% confidence interval to assess precision of the estimates, and a *P* value ≤ 0.05 was considered statistically significant.

One can confuse this "n" with the "n" of sample size. Better to remove this "n".

Results: The prevalence of hepatitis B and C infections among the 6085 female sex workers was 2.6% [(95% CI (2.2,2.8)] and 0.5% [(95% CI (0.4,0.7)], respectively. Female sex workers who had 61-90 and ≥91 paying clients in the past six months [(AOR=1.66; 95% CI, (0.99, 2.79); *P*=0.054] and [(AOR=1.66 95% CI, (1.11, 2.49); P=0.013], respectively, age at first sex selling of 20-24 and >25 years [(AOR=1.67; 95% CI, (1.14, 2.44); *P*=0.009)] and [(AOR=1.56; 95% CI

Be consistent: Commercial female sex workers or female sex workers.

How can you make sure that females sex workers have 61-90 even more clients?

This sentence is not completed.

The result part is not clear. Authors should make sure that the message is fluent.

The direction of the comparison should appear in the text.

(1.004, 2.43); *P*=0.048)], respectively, known HIV positive status [(AOR=1.64; 95% CI (1.03, 2.62); *P*=0.036] were significantly associated with the prevalence of hepatitis B virus infection. Similarly, hepatitis C was significantly associated with, age at first sex ≤15 years and age 16-20 years [(AOR=0.21; 95%CI (0.07,0.61); *P*=0.005)] and [(AOR=0.18; 95% CI (0.061, 0.53); *P*=0.002)], respectively, known HIV positive status [(AOR=2.85; 95%CI (1.10,7.37); *P*=0.031)] and testing positive for syphilis [(AOR=4.38; 95% CI (1.73,11.11); *P*=0.002)], respectively.

Conclusion: This analysis reveals an intermediate prevalence of hepatitis B and a low prevalence of hepatitis C infection among female sex workers in Ethiopia. It also suggests that population groups with like female sex workers are highly vulnerable to hepatitis B, hepatitis C, and other sexually transmitted infections. There is a need for strengthening treatment and prevention interventions, including immunization services.

Which immunization? Be specific for better intervention.

Keywords: hepatitis B, hepatitis C, female sex workers, syphilis. Ethiopia

Key words not at this place.
Editors will handle it.

INTRODUCTION

Viral hepatitis is an important public health problem globally, and hepatitis B virus (HBV) and hepatitis C virus (HCV), in particular, are endemic in developing countries (1). It is estimated that 325 million people were living with HBV and HCV in 2019 globally (2). In 2013, viral hepatitis was the seventh-highest cause of mortality in the world and it was responsible for an estimated 1.4 million deaths per year, mostly from hepatitis-related liver cancer and cirrhosis; approximately 47% of the deaths were attributable to HBV, 48% to HVC, and the remainder to hepatitis A and E infections (3). According to the 2017 Global Hepatitis Report, viral hepatitis in general caused 1.34 million deaths in 2015, a figure that was higher than the deaths caused by HIV infection and comparable with that caused by tuberculosis (4).

Seventh-highest: What is the comparison?

In the African Region, HBV infection is highly endemic and affects an estimated 5%-8% of the population, mainly in West and Central Africa (5). People living with HIV (PLHIV) are at high risk of becoming ill and dying from hepatitis. Some 2.6 million PLHIV are co-infected with HBV,

WHO: used at the first time, authors should spell it.

and some 2.3 million with HCV (6). The WHO 2019 Progress Report on HIV, viral hepatitis, and sexually transmitted infections (STI), showed, in the sub-Saharan Africa (SSA), there were over 60 million cases of chronic hepatitis B and over 10 million cases of chronic hepatitis C infections (7).

In SSA, female sex workers (FSW) had a high-risk behavior and remain important in terms of transmission of HBV, HCV, HIV/AIDS and other STI acquisition and transmission. This could be because FSW have numerous sex partners and they were engaged in unprotected and other forms of sex that cause contact with body fluids of a partner who has STI. FSW are often in a weaker position to negotiate safe sex because of social, economic, cultural and legal reasons (8).

Commercial sex work (CSW) is a high-risk activity associated with HBV, HCV, and several other STI (6, 9). The higher risk of getting infected with HIV and other STI, such as syphilis and hepatitis among FSW is primarily associated with the high number of sexual partners and increased

Redundancy. This was said in the above paragraph, line 73

frequency of unprotected sex. Several studies have shown that low adherence to condom use, multiple sexual partners, unsafe sexual practices, illicit drug use, and co-infection with other STI

Ref

increase the risk of HBV and HCV transmission. FSW also have a higher risk of contracting STI from their non-paying partners than from their paying clients (10,11).

Ref

In Ethiopia, FSW carry a disproportionate burden of HBV, HCV, and HIV infection. According to the Ethiopian Demographic and Health Survey (EDHS) 2016 report, the marked regional variation that was driven by most at-risk populations (MARPS) indicates that urban areas and females are more affected than rural areas and males, respectively (12). Small towns are also

becoming hotspots and can potentially bridge further the spread of the HIV and HBV infections to rural settings, where the female are twice more affected than males (12).

Ethiopia is in the region where HBV prevalence is considered hyper-endemic with a prevalence of between 8%-12%, and that of HCV prevalence is estimated at not less than 2.5% (13). An earlier study conducted in Ethiopia reported that 12% of hospital admissions and 31% of the mortality on the medical wards in Ethiopian hospitals were due to chronic liver disease (CLD) (13). However, there isn't much done and the available data on associated chronic liver disease or hepatocellular carcinoma are not sufficient.

Do you mean that HBV among FSW is less than the HBV among the general population in Ethiopia?

There are limited data from isolated studies showing the prevalence of HBV in Ethiopia - Hawassa 9.2% (14), Gonder 28.9% (15), Mekelle 6% (16), Dessie 13.1% (11), and northwest Ethiopia (11.9%) (17). FSW have been identified as a population group with the highest risk for STI, including HBV and HCV, and should perceive priority in the national HIV/AIDS program (18). Although there is an ongoing HBV, HCV, and other STI program in the country, there is no national data among FSW to determined HBV and HCV prevalence and driving factors. Therefore, the current study was conducted to explore the prevalence of HBV and HCV infections and identify the factors associated with these infections among FSW in Ethiopia.

Where these mentioned studies were conducted and in which group population?

MATERIALS AND METHODS

Study setting and population:

The study was done in Ethiopia, a country divided into eleven regions and two city administrations, and with a population of 119.8 million, some 80 ethnic groups, and a land area of 1.0 square kilometers, and an average population density of 121 inhabitants per square kilometers (19). It had a gross domestic product growth of 6.1% in 2020 and a per capita income of USD 850 (20). The median age of the population is 19.6 years, population growth rate 2.56%, total fertility rate (TFR)

4.6, infant mortality rate (IMR) 41per 1,000 live births, and adult literacy rate of 49% in 2016 in 2016, when the country started a national viral hepatitis prevention and control program (21). Regional capital cities and selected towns with the highest number FSW (hotspots), including Adama, Addis Ababa, Arba Minch, Bahir Dar, Combolcha/Dessie, Dilla, Dire Dawa, Gambella, Gonder, Harar, Hawassa, Jimma, Logia/Semera, Mizan, Nekemite, and Shashemane were involved in the study.

The information of Ethiopia background should be summarized and focus on settings where the study have been conducted.

Study design and period:

cross

This was a crass-sectional, nation-wide, biobehavioral study conducted among FSW aged ≥15 years during the period from December 2019 - April 2020.

Target population:

The target population of the study is all FSW living in cities and towns in Ethiopia, and the sampling frame is the list of FSW residing in the regional capitals and selected towns with FSW-hotspots in Ethiopia.

1. How towns and cities were selected?
2. There are no FSW in rural settings?
3. It is not national representative study because some areas were excluded.

Study population:

FSW aged ≥15 years residing in regional capitals and selected towns with FSW-hotspot or who worked in these cities and towns in the last one month preceding the survey. The survey included both fixed (venue-based) and floating (street-based) FSW.

Inclusion and exclusion criteria:

We included women aged ≥15 years, who received money/other benefits in exchange for sex with four or more people within the last 30 days, agree to participate in the survey including interviewing and biological testing, able to provide informed consent and communicate in one of the survey languages, had a valid coupon provided by the study team , and residing or working in the survey city or town for the last one month.

Sample size and sampling procedure:

The sample size was determined by single population proportion formula

$$N_t = \left[\frac{Z^2 p(1-p)}{d^2} \right] * DEFF$$

The formula is not needed in manuscript writing. Better to delete it

using 95% confidence interval, α = 0.05, margin of error of 35% (d), and proportion (p) (22) of 2%, and DEFF = design effect with a replacement for non-responders. With these assumptions, the minimum desired sample size of FSW in sixteen (n=16) major regional capitals and selected towns with FSW hotspots was 6085 after adding 10% contingency. This was divided and assigned to 16 sites proportionate to population size.

What is needed is not the calculation of cities rather the calculation of the size of participants (FSWs). Cities should according to existing hot spots.

Specific hotspot areas for FSW were identified during tools and procedures pretesting with support from HIV/AIDS Prevention and Control office (HAPCO), woreda (district) health offices, and drop-in-clinics (DICs), local organizations working with FSW. We used a respondent-driven consecutive sampling using a standardized questionnaire for recruitment of study participants. The local organizations assisted in identifying the initial respondents of the survey, referred to as "seeds". The number of seeds for each site was determined based on the result of a formative assessment. Five "seeds" for each site with allocated sample of <450, six-eight seeds for each site with sample 450-900, and 12 seeds for each site with sample of 1101 were recruited. The "seeds" were selected based on the type of sex worker, age category, and geographic location of the site. These include those FSW who were bar- and/or hotel-based, red lighthouses, local drinking houses, street-based and hidden (cell phone-based).

FSW with a known social network were given each three coupons for use to invite her friends or other FSW contacts who were in her network. This approach helped in reaching as many eligible FSW as possible. The coupon remained active from the day it was given to the potential participant

and expired after two weeks or if the study was completed earlier. We used anonymous fingerprint-based code obtained using biometric fingerprint scanners to ensure that all respondents participated only once. This was not linked to the biobehavioral questionnaire and was used only for avoiding multiple enrollments.

Damaged, mutilated, not readable, photocopied, not sealed/stamped coupon was considered not valid. Each participant coming to the study site would need to bring her coupon that was identified by specific number given by the referring person. New participants were given coupons and asked to recruit three additional acquaintances. This process continued until the desired sample size was achieved and respondent-driven sampling (RDS) equilibrium condition attained. Progress towards reaching the equilibrium was monitored by using key parameters including current HIV status, type of sex work, and consistent condom use.

How a new participant coming without a coupon could be enrolled?
How many waves have you registered?
Number of waves stopped before the end of the process.

Data collection procedure and data quality management:

Data were collected using a pre-tested structured questionnaire initially developed in English and then translated into the local language (Amharic) was entered onto open data kits (ODK) software. Training was provided to the study team, coordinators, interviewers, blood sample collectors, coupon managers (for RDS), receptionists (for RDS) and accompanying referral liaisons. The training included different topics with a focus on the survey sampling methodology, procedures, and data collection tools, and overall study site management. Data collection tools and questionnaires were pretested in a pilot survey in Bishoftu town, a site not included in the main study. Feedback from the pilot was used to finalize the data collection tools, capturing process, field logistics, and operational procedures. The survey used whole blood for the rapid HIV, hepatitis-B and syphilis testing. After collecting whole blood of 5 ml using EDTA tube; HIV testing, Hepatitis B surface antigen (HBsAg), hepatitis C antibody (HCVAb) and syphilis testing

were performed right after sample collection. Then after centrifuging the whole blood, the plasma was separated and a liquated in two a 1.8 ml preprinted labeled nunc tube for viral load quantification and quality control testing.

- Authors should specify the name of tests used in the study.
- Test algorithm
- Sample storage and transportation.
- Clinical management once the test is positive
- Syphilis and HIV results are not part of the study.

Biological analysis:

HBsAg and HCVAb were screened by using a rapid test kit according to manufacturer principles and procedure. Testers were trained on how to use the test kit and control lines. All invalid results were repeated. Quality control (QC) panels consisting of a positive and negative control specimen were done in parallel with the testing procedure to ensure test kits were performing correctly. Other STI, including HIV and syphilis, were tested using the national rapid testing algorithm and the results were returned during the second study visit. Syphilis was screened using Chembio Dual Path Platform (DPP) Syphilis Screen and Confirm Assay, according to manufacturer principles and procedures in the kit insert.

Data Analysis:

The data was collected using the ODK software on tablet computers, and was exported to MS-EXCEL, cleaned, and imported to STATA Verssion16 for analysis. The RDS recruitment process (Tree of recruitment), assessment of the RDS assumptions, and RDS weight generating were implemented using the RDS package inbuilt in R statistical software (23). Homophily and convergence, the common assumptions in RDS, were checked in HIV status, consistent condom use, and type of FSW and met the RDS criteria. The RDS weights were exported using the RDS-II function to STATA and merged with the whole dataset for further analysis. Descriptive statistics like the crude and RDS adjusted frequency, mean and standard deviation were calculated. Bivariate and multivariate logistic regression analyses were conducted to determine the strength of association between independent variables (risk factors) and the outcome variables (hepatitis B

In the abstract section authors mentioned that <=0.05 p-value was considered. 0.2 p-value is too high, it can be considered in some few key variables.

and C virus infection). Variable achieving a *P* value of <0.2 in the bivariate analysis were included in the multiple logistic regression model. Strength of association was measured using Adjusted Odd Ratios (AOR), precision of estimates determined using 95% confidence intervals, and a *P* value ≤ 0.05 was used as cut-off to determine statistical significance.

Ethical Consideration:

Ethical approval for the study protocol was obtained from the Scientific and Ethical Research Office (SERO) of the Ethiopian Public Health Institute (EPHI). Potential participants were told about the study purpose and procedures, potential risks, and protections using the local language. Written informed consent was obtained from each survey participant for the interview, blood sample collection, and storage of biospecimens for future testing.

No incentive was provided to participants?

RESULTS

Socio demographic characteristics:

A total of 6085 FSW participated in the survey. Their median age [Interquartile Range (IQR = 8] was 25 years, and the highest number, 1980 (32.5%), were in age group 20-24 years and the lowest 615 (10.1%) in the age group 15-19 years (Table 1). A majority, 5031(82.7%), of them had formal education, 2946 (48.4%) were never married, and 4212 (69.2%) were pregnant a least once. A majority, 5694 (93%), of the respondents reported that selling sex was their main source of income, and 2066 (34%) of them had an average monthly income of Ethiopian Birr (ETB) 2500-4999 equivalent of U.S Dollar (USD) 60 – 120.

Median=25 and IQR=8? Check please.

Table 1: Socio-demographic characteristics among female sex workers in cities/towns, Ethiopia, 2020 (n= 6085)

The table should be formatted for the clarity of the reader.

Variables		Frequency	Percent
Age (years), Median (IQR)	25 (8)		
Age in Year	15 – 19	615	10.1
	20 – 24	1980	32.5

	25 – 29	1815	29.8
	30 – 34	856	14.1
	35 – 59	819	13.5
Level of education	Non-formal Education	1054	17.3
	Primary 1st cycle (grade 1-4)	848	13.9
	Primary 2nd cycle (grade 5-8)	2712	44.6
	Secondary school and above	1471	24.2
Marital status	Married/Cohabitation	231	3.8
	Divorced/Separated/Widowed	2908	47.8
	Never married	2946	48.4
Ever been pregnant	No	1873	30.8
	Yes	4212	69.2
Number of pregnancies	0	1873	30.8
	1	2059	33.8
	2	1238	20.3
	3+	915	15
Ever been pregnant	No	1873	30.8
	Yes	4212	69.2
Number of pregnancies	0	1873	30.8
	1	2059	33.8
	2	1238	20.3
	3+	915	15
Currently pregnant	No	5973	98.2
	Yes	112	1.8
The main source of income	Other than sex work	391	6.4
	Sex work	5694	93.6
Average monthly income from selling sex (ETB)	< 2500	1778	29.2
	2500 – 4999	2066	34
	5000 – 7499	1175	19.3
	7500+	1066	17.5

Distributions of FSW by study cities/towns:

18.1% is not the majority.

A majority, 1101 (18.11%), of the 6085 FSW resided in Addis Ababa, the national capital followed by Adama, 676 (11.1%). A smaller proportion of them were in other cities/towns (Table 2).

Table 2: Distributions of female sex workers by cities/town

in Ethiopia, 2020 (n= 6085)

**Residence (City/Town)**	**Frequency**	**Percent**
Adama	676	11.1
Addis Ababa	1101	18.1

Hawassa	522	8.6
Gambella	468	7.7
Diredawa	434	7.1
Bahir Dar	372	6.1
Jimma	254	4.2
Mizan	255	4.2
Nekemite	257	4.2
Arba Minch	251	4.1
Combolcha/Dessie	251	4.1
Dilla	251	4.1
Diredawa	434	7.1
Gambella	468	7.7
Gonder	250	4.1
Logia/Semera	251	4.1
Shashemane	250	4.1
Harar	242	4.0

Sexual and behavioral characteristics:

Review the meaning of IQR

The median (IQR) age at first sex was 16 (3) years. The majority, 3384 (55.6%), of respondents had the first sex between the age of 16 and 20 years (Table 3). Some 2335 (38%) of the respondents had first sex with a person of their age, while 2425 (40%) of them had first sex with persons older by 5-10 years. The sexual practice was in hotels and bars in 2023 (33.2%) and was street-based in 1871 (30.7%). Alcohol dependence was report 2257 (37.2%) and chewing chat in 3827 (62.4%) of respondents during the last thirty days before the survey.

Condom use was practiced in 5119 (84%) FSW. Of the remaining 966 (16%) who were not using condom, 23 (2.4%) were HBsAg positive, and among those who reported using condoms systematically at every sexual intercourse, 134 (2.7) were HBsAg positive. HCVAb was positive in 4 and 23 of those who used condom consistently and inconsistently, respectively.

The first sex experience of the FSW was in 1347 (22.1%) forced, whereas in 4738 (77.9%) not forced. In the last six months preceding the study, 1645 (27%) of the FSW reported they had more

than 90 clients. The majority, 5119 (84.1%), of the FSW utilized a condom during sex, and condom breakage was experienced by 1825 (30%).

Table 3: Sexual and behavioral characteristics of female sex workers in cities /towns, Ethiopia, 2020 (n= 6085)

Variables		Frequency	Percent
Age at first sexual intercourse (years), median (IQR)	16 (3)		
Age at first sexual intercourse (years), n (%)	≤15	2430	39.9
	16-20	3384	55.6
	≥21	271	4.5
Age at first sex selling	< 20	2328	38.3
	20-24	2348	38.6
	≥25	1406	23.1
Age of first sex partner	5 or more years younger	73	1
	About the same age	2335	38.4
	5-10 years older	2425	39.9
	More than 10 years older	1252	20.6
Location of sexual practice	Hotel/bar-based	2023	33.2
	Street-based	1871	30.7
	Home-based	348	5.7
	Any type**	661	10.8
First sex experience	Wanted	4738	77.9
	Forced	1347	22.1
Number of paying partners in the last 6 months, n (%)	≤ 30	2308	37.9
	31 – 60	1436	23.6
	61 – 90	696	11.4
	≥91	1645	27
Ever had Anal intercourse, n (%)	Yes	426	7
	Never	5659	93
Used condom during anal sex (n=426)	Yes	252	59.2
	No	174	40.8
Consistent Condom utilization during the last 30 days with paying clients	Yes	5119	84.1
	No	966	15.9
HBV among non-condom users during the last 30 days with paying clients	HBsAg positive	23	2.4
	HBsAg negative	943	97.6
HBV among condom users during the last 30 days with paying clients	HBsAg positive	134	2.7
	HBsAg negative	4651	97.3
Breakage of condom during the last 30 days	No breakage	4260	70
	Experienced breakage	1825	30
In the last 30 days, used cigarettes or cigars	No	5343	87.8
	Yes	742	12.2
Alcohol consumption level (AUDIT scores)	Not Risky	2594	42.8
	Harmful, hazardous drinking	1210	20
	Alcohol dependence indication	2257	37.2
Chewing khat in the last 30 days	Yes	3827	62.9

	No	2258	37.1
Any drug used other than alcohol and khat in the last 30 days	Yes	700	11.5
	No	5382	88.4
Used shisha in the last 30 days	Yes	856	14.1
	No		85.9

*Alcohol Use Disorders Identification Test (long version)

**Any type: Restaurant/cafe /cake bet, Local drink house (arake bet, tella bet, tej bet), SPA/massage/beauty, Redlight

Prevalence of co-infection of HBV, HCV, syphilis and HIV among FSW:

Of 1,140 FSW infected and 4945 not infected with HIV, 40 (3.5%) and of 117 (2.2%) were coinfected with HBV, respectively (Table 4). HCV coinfection was documented in 12 (1.1%) of those infected and 15 (0.3%) in those not infected with HIV. Syphilis was diagnosed in 339 (3.6%) of the FSW, and HBV and HCV coinfections with syphilis were seen in 3.8% and 2.1% among those infected and not infected with syphilis, respectively.

This statement is not clear. HBV co-infection with HIV and not HIV?

The same as above. If not HIV infected you cannot say co-infection.

Why is syphilis reported in this study?

Table 4: Weighted prevalence of hepatitis B and hepatitis C co-infection with syphilis and HIV among female sex workers, Ethiopia, 2020 (N = 6085)

HBV, HCV and HIV prevalence should be displayed by social dem char

Variables		Frequency	Percent
Depression level	Not depressed	2468	40.6
	Mild depression	2525	41.5
	Moderate to severe depression	1092	17.9
History of STI infection	Syphilis	339	5.6
	Abnormal vaginal discharge	887	14.6
	Syphilis HIV co-infection	161	2.6
	Genital ulcer	381	6.3
HIV test result	Tested negative	4945	81.3
	Tested new positive	565	9.3
	Already known positive	575	9.4
Hepatitis B/HIV co-infection	HIV negative	117	2.2
	HIV positive	40	3.5
Hepatitis C/HIV co-infection	HIV negative	15	0.3
	HIV positive	12	1.1
Syphilis/HBV and HCV co-infection	HBV co-infection	13	3.8
	HCV co-infection	7	2.1

Prevalence of HBV and HCV

Of the 6085 FSW tested for HBV, 157 were tested positive for the HBsAg, a prevalence of 2.6% (95% CI (2.2, 2.8), and 27 FSW were positive for HCVAb, a prevalence of 0.5% (95% CI (0.4, 0.7). Only 5 (0.1%) of the participants were co-infected with both HBsAg and HCVAb. Among the 6085 participants in the study, a total of 184 (3%) were infected by HBV and HCV (Table 5).

Table 5: Weighted Prevalence of hepatitis B and hepatitis C infections among female sex workers, Ethiopia, 2020 (N = 6085)

Not clear about the HBV and HCV co-infection. 0.5% or 3%? 0.09% in the table below.

Not characteristic instead infections

**Characteristic**	**Overall (N=6085)**	**Percent (%)**	**95% CI**
HBV	157	2.6	2.2-2.8
HCV	27	0.5	0.4-0.7
HBV/HCV	5	0.09	0.03-0.29
HBV among non-condom users during sex (n=966)	23	2.4	2.0-3.4
HBV among condom users during sex (n=5119)	134	2.6	2.2-2.8)
HCV among non-condom users during sex (n=966)	4	0.4	0.2-0.8
HCV among condom users during sex (n=5119)	23	0.5	0.4-0.7

Factors associated with hepatitis B and C:

Results of bivariate and multivariate logistic regression analyses are presented in Table 6. In the bivariate analysis, FSW with HBV infection had a significantly higher odds of being in the age groups 25-29 and 30-34, [COR=1.2; 95% CI (1.01, 3.93), *P*=0.045)] and [COR=2.35; 95% CI (1.14, 4.81), *P*=0.02)], respectively, compared with the age group 15-19 years. The odds of having over 90 sexual partners compared with those having under 30 partners in the past six months of being in age groups 20-24 or 25 years and above at first sex selling compared with those under 20, and of being HIV positive compared with being HIV negative was significant among FSW with HBV infection, [COR=1.6; 95% CI (1.07, 2.4), *P*=0.072)], [COR=1.68; 95% CI (1.15, 2.46), *P*= 0.008)], [COR=1.64; 95%CI (1.06, 2.52), *P*=).025)] and [COR=1.72; 95% CI (1.09, 2.71), *P*=

0.020)] respectively. In the multivariate logistic regression analysis, having more than 90 sexual partners in the past six months compared with those having less than 30 partners, being in the age groups 20-24 and ≥25 at first sex selling compared with those under 20 years, and being HIV positive, [AOR=1.66; 95% CI (1.11, 2.49), *P*= 0.013)], [AOR=1.67; 95% CI (1.14, 2.44), *P*= 0.009)], [AOR=1.56; 95% CI (1.004 2.43), *P*=0.048)] and [AOR=1.64; 95% CI (1.03, 2.62), *P*=0.036)], respectively, are significantly and independently associated with HBV infection among FSW.

In the bivariate analysis, FSW with HCV infection had a lower odds of being in the younger aged group of 15 years or under was significantly higher [COR=0.24, 95% CI (0.08,0.70), *P*= 0.009)] and 16-20 years [COR=0.17; 95% CI (0.06,0.50), *P*= 0.001)] compared those aged above 20 years. The odds of being newly HIV positive or known HIV positive compared with being HIV negative and being positive for syphilis compared with being negative for syphilis was significant [COR=2.93; 95% CI (1.06-8.10), *P*= 0.038)], [COR=4.05; 95% CI (1.64-9.98); *P*=0.002)] and [COR=6.03; 95% CI (2.53, 14.38), *P*= 0.00)], respectively. In the multivariate logistic regression analysis, FSW with HCV infection is significantly and independently associated with age at first sex of 15 years or less [AOR=0.21; 95% CI (0.07, 0.61), *P* = 0.005)] and age 16-20 years, compared with age of 25 years and above [AOR=0.18; (0.061, 0.53), *P*= 0.002)] known HIV status [AOR=2.85; 95% CI (1.10, 7.37), *P* = 0.031)] and being positive for syphilis [AOR=4.38; 95% CI (1.73,11.11), *P* = 0.002)]

Table 6: Factors associated with hepatitis B and hepatitis C among female sex workers, Ethiopia, 2020

Variable	Frequency	HBV (n=157)				HCV (n=27)			
		COR (95%C.I)	*P*-value	AOR (95%CI)	*P*-Value	COR (95%C.I)	*P*-value	AOR (95%CI)	*P*-value
Age	15 - 19	1*							
	20 - 24	1.15 (0.57, 2.33)	0.390						
	25 - 29	1.2 (1.01, 3.93)*	0.045						

	30 - 34	2.35 (1.14, 4.81)*	0.02						
	35 - 59	1.51 (0.7, 3.26)	0.3						
Number of sexual partner in the past 6 months	< 30	1*							
	31 - 60	1.3 (0.84, 2.02)	0.240	1.31 (0.84, 2.03)	0.228				
	61 - 90	**1.6 (0.96, 2.69)***	**0.072**	**1.66 (0.99, 2.79)****	**0.054**				
	91+	**1.6 (1.07, 2.40)***	**0.021**	**1.66 (1.11, 2.49)****	**0.013**				
Average monthly income from selling sex in ETB	< 2500	1*							
	2500 - 4999	1.16 (0.76, 1.77)	0.496						
	5000 - 7499	1.49 (0.94, 2.36)	0.089						
	7500+	1.37 (0.85, 2.22)	0.198						
Moderate to severe depression	Not depressed	1*							
	Mild Depression	0.72 (0.51, 1.02)*	0.067			0.98 (0.39,2.47)	0.961		
	Moderate to severe depression	0.85 (0.55, 1.32)*	0.474			2.27 (0.90-5.74)	0.083		
Number of cities worked sex selling in the last three years	Same town								
	1 more town	1.48 (0.97, 2.27)	0.069						
	2 or more towns	1.48 (0.83, 2.66	0.186						
Number of non-paying partners in the past 6 month	Never								
	Only one	2.34 (0.86, 6.38	0.096						
	2 and more	1.98 (0.70, 5.64	0.198						
Age at first sex	15 or less	??	??	??	??	0.24 (0.08,0.70)	0.009	**0.21 (0.07,0.61)****	**0.005**
	16 - 20	??	??	??	??	0.17 (0.06,0.50)	0.001	**0.18 (0.061,0.53)****	**0.002**
	21+					1*			
Age at first sex selling	< 20	1*							
	20 – 24	**1.68 (1.15, 2.46)**	**0.008**	**1.67 (1.14, 2.44)****	**0.009**	0.59 (0.22,1.64)	0.314		
	25+	**1.64 (1.06, 2.52)**	**0.025**	**1.56 1.004, 2.43)****	**0.048**	1.83 (0.77,4.31)	0.169		
HIV Test Result	Tested Negative	1*							
	Tested New Positive	1.28 (0.76, 1.14)	0.349	1.249 (0.74, 2.09)	0.410	2.93 (1.06-8.10)	0.038	2.05 (0.71,5.92)	0.186
	Known Positive	**1.72 (1.09, 2.71)***	**0.020**	**1.64 (1.03, 2.62)****	**0.036**	**4.05 (1.64-9.98)***	**0.002**	**2.85 (1.10,7.37)****	**0.031**
Syphilis	Non-Reactive	1*							
	Reactive	1.55 (0.87, 2.77)	0.137			**6.03 (2.53-14.38)**	**0.000**	**4.38 (1.73,11.11)**	**0.002**

1*- reference (used as constant); *- significant on Bivariate analysis; **- significant at multivariate analysis

1. Consistency in data analysis:
- Age group categories with p-values <0.05 not appearing in multivariable model. The same for number of sexual partners
2. Multivariable model without bivariate model. What happened? Age of first sex
3. Age at first sex selling not completed

DISCUSSION

The discussion section is not structured.
The author is reporting the results
The reader will be bored with this so long and light discussion

Of the 6085 FSW enrolled in our study in 2020, the prevalence of HBV among FSW was 2.6% and of HCV was 0.5%, which are comparable with a similar, previous study. Based on the WHO HBV infection prevalence classification - high (>8%), intermediate (2-8%), and low (<2%) (24), HBV infection prevalence in Ethiopia is the intermediate category. This translates to almost one in every thirty-seven FSW having HBV infection. The prevalence we have identified in the Ethiopian setting is lower than the prevalence reported from the rest of Africa and South-East Asia (5%), but higher than the prevalence in the Americas and Eastern Mediterranean (1%). Ref This contributes to the 1.5 million new HBV infections occurring globally each year and will have major implications to the epidemiology of chroming HBV infection; nearly 3.6% of the world's population (257-296 million) have chronic HBV infection, a chronic HBV infection prevalence of 0.01-2% in the United Kingdom, United States, Canada, Western Europe, and Japan, and over 8% in most SSA and Western Pacific regions (25-28). Ref

In countries with high and middle-income, HBV transmission is more perinatal and horizontal, whereas, in low-income countries, the transmission occurs through drug injection and high-risk sexual behaviors (29). Africa and Asia have the highest endemicity of HBV, though effective vaccination programs have pushed the burden towards moderate or low endemicity Most countries in Africa have high endemicity, with the exception of Tunisia and Morocco, which have moderate endemicity (30,31). The WHO global hepatitis strategy aims to reduce new hepatitis infections by 90% and deaths by 65% between 2016 and 2030 (32), and according to WHO, 80% of people with hepatitis live without prevention, testing, and treatment of HBV infection (33).

There is much variation in the prevalence HBV infection across countries, including those which have a lower prevalence than ours like Mexico 0.2% (34), Iran 1.1% (35), Greece 1.3% (36), Brazil

0.7% (37), those with similar prevalence to ours like Rwanda 2.5% (38), and countries in SSA having much higher prevalence than ours like Nigeria 17.1% (39), Kenya, 13.3% (40), and Ghana 15.0% (41). Similarly, isolated and limited studies on HBV conducted in different cities/towns in Ethiopia at various times reported varying prevalence by site and year of study - Dessie 13.1% (11), Hawassa 9.2% (14), Gonder 28.9% (15), and Mekelle 6% (16). This variation could largely be explained by the differences in sociodemographic characteristics of study populations, study settings, sample size, and sampling methods focusing on high-risk population groups.

Our study showed that HBV prevalence was significantly associated with the age groups 25-29 years and 30-34 years compared to the age group 15-19 years in the bivariate analysis, but these

If not significant, why to discuss it?

did not achieve significant independent association in the multivariate analysis (P > 0.05). This is consistence with the finding by Forbi JC. et al (39) who reported that, although the prevalence of HBV is highest among FSW in the 30-35 years age group, it is not statistically significant. In contrast to this finding Vázquez-Martínez, et al (42). Recognized that being above 30 years of age

How can you exclude the cohort effect in this case?

is significantly associated with HBV infection. the duration of staying as CSW among FSW increased the infection risk of HBV with increased age. In Our study only individuals above 15-years were recruited. the finding implies awareness creation and high engagement of health workers will be needed to vaccinate high-risk groups like FSW.

This statement is not contributing in this section.

Having more than 90 sexual partners in the past six months on a monthly average of 15, compared with those having less than 30 partners in the past six month, almost one partner on monthly

You come again in results section not discussion.

average was independently associated with HBV infection. The positive association of HBV exposure with having a greater number of clients indicates that FSW were vulnerable to infection through sexual risks. This is not surprising as sexual transmission is an important route of transmission of HBV infection. Generally speaking, the reported average number of clients who

had a history of multiple sex partners may increase the probability of having sex with an infected partner during the acute phase of infection this study was in agreement with study conducted by the WHO 2014 Global Network of Sex Work Projects, Prevention and treatment of HIV and other STS for sex workers in low and middle-income countries and study result from Ethiopia, India, Brazil, Egypt and Japan (43-49).

Being in the age groups 20-24 and 25 years and above at first sex selling was significantly and independently associated with HBV infection compared with those under 20 years. This finding was inconsistency with study reports from Nigeria and Mexico, which support early age of sexual activities increases the risk of HBV infection (39,42). This variation might be an indication for FSW in this study had sexual involvement at an early stage and did not get HBV vaccination during their childhood because the national viral hepatitis prevention and control program was initiated in 2016 in Ethiopia. Thus, most of our study participants did not have the opportunity for early vaccination. However, in the previous studies conducted in Nigeria and Mexico, HBV vaccination

This sentence is not contributing.

was given in early childhood. In addition, the females in Ethiopia are economically independent of their families at the age of 20 years or above. In order to increase their daily expenses the FSW might be sexually active and engaged with many partners which increases their exposure to HBV.

We could not find any report that indicated the significant association of being HIV positive with

You can find such studies.

HBV infection alone. However, the study reported from Rwanda on Syphilis and HIV prevalence and associated factors to their co-infection among FSW indicated that HBV infection is an

Syphilis is not included in your study.

independent predictor of HIV and syphilis co-infection, and the odd of having an HBsAg-positive test is 2.09 times higher in syphilis/HIV co-infection FSW(38). This result was almost similar to our finding in which Being HIV positive compared with being HIV negative was significant among FSW was independently associated with HBV infection in which being HIV positive was

1.64 times more likely to be infected by HBV than FSW who were HIV negative. The presence of HBV infection among FSW might be due to a higher risk of developing hepatotoxicity following the initiation of antiretroviral therapy or a lower CD4 T-cell count.

Not sure. HBV is an STI

You reported 3 values of HCV.

We found that the overall prevalence of HCV among FSW was 0.5%, a finding similar to that reported form Nairobi, Kenya (0.76%) (43), and a previous study in Ethiopia (0.7%) (50). Our finding is also concurs with the prevalence ranging between 0-1.4% reported from the united States and Europe (51,52) as well as the global average prevalence of HCV infection (0.8%) (53). In contrast our finding is lower than the prevalence reported from Iran of 6.2 % (54), Ghana of 2.8% (55), and Port Harcourt, Nigeria 2.9% (56). Similarly, syphilis seroprevalence of 5.6% from this study is higher than that of Ghana 7.5% (57), Tanzania 12.7% (58), and a previous report from Ethiopia 1.3% (50). Our finding indicates that the prevalence of HCV was relatively lower compared to similar finding reported elsewhere. The reason for the lower report might be the improvement in technology where the current screening reagent to be more specific and reliable, and could also be an indication for the improvement of the national prevention or a treatment programs for HCV. It could also be an indicator that there are geographical discrepancies in prevalence of HCV infection.

Our finding indicates, that FSW with HCV infection had a lower odds of being in the younger aged group of 20 years or under is significantly and independently higher compared to those aged above 20 years. The rise in anti-HCV positivity with age in this group might be the result of continuous exposure to the virus. Our finding suggests that HCV seroprevalence is lower among the younger FSW and this might be a reflection of lower exposure to sexual risk behaviors and the small sample size in the younger age group which need be taken into consideration, Concurring

with our finding, a previous study also indicated that the low HCV seroprevalence in younger CSW (59).

Mainly because of the nature of HIV route of transmission, many HBV or HCV-infected individuals were co-infected with HIV. Our study adds to the evidence showing that co-infection of HIV and viral hepatitis occurs frequently largely because of their mode of transmission. The odds of being newly diagnosed with HIV positive or having a known HIV positive status among FSW with HCV infection was significantly and independently higher compared with HIV negative FSW with HCV. FSW who were known HIV positive were 2.85 times at higher risk of being HCV infected compared to those who were HIV negative. This finding was supported with a study conducted in Burkina Faso, West Africa, and the results of a systematic review on HIV and the hepatitis virus co-infection in studies from SSA, which reported that those who were HIV infected were estimated to be at higher risk of HCV coinfection compared to HIV seronegative people (AOR = 5.59) and (RR=1.60), respectively (17,60). The hepatitis C virus is usually spread when someone comes into contact with blood from an infected person. This can happen through sharing drug injection equipment, though drug injection is uncommon in Ethiopia (61). Our finding suggest that the route of transmission for HCV among sexually active individuals is commonly sexual. After stratification by HIV status, HCV prevalence among women of the general population was identical to that of FSW, suggesting that HCV sexual transmission is not common in this population and that HIV infection does not enhance susceptibility to HCV sexual transmission (62). However, the significant association between HCV and HIV infection among FSW has not been clearly described in published studies and this needs further investigation.

The odds of being positive for syphilis compared with being negative was significantly and independently associated with being HCV positive among FSW. In agreement with our finding, a

previous study also indicated that HCV sero- reactivity or positivity was significantly predicted and associated with syphilis sero- reactivity or positivity (63). Moreover, a study conducted by Tessema B, et al (50) suggested that the highest rate of co-infection and the statistically significant relationship between HCV and syphilis infections might be due to the fact that these pathogens share common modes of transmission and risk groups. These findings are in line with our finding showing that FSW with HCV infection were 4.4 times more likely to be syphilis sero-reactive or positive than those who were syphilis non-reactive or negative. As HCV positive FSW were at a higher risk for having syphilis, prevention mechanisms and intervention need to be instituted among FSW to decrease further transmission of HCV and syphilis to the general population.

Of 6085 participants included in our study,184 (3%) had results for both HBsAg and HCVAb. The prevalence of HBV/HCV, HIV/HBV, and HIV/HCV co-infection among HIV positive FSW in the present study was 3.6%, 1.3%, and 0.09%, respectively. A similar finding was reported from the Global Prevalence of HBsAg and HIV and HCV Antibody study, which showed that the prevalence of HIV/HBV and HIV/HCV co-infections among FSW was 3% and 1%, respectively (31).

The previous prevalence estimates of HBV among general population in Ethiopia ranged from 8%-12%, and HCV prevalence estimated at greater than 2.5% (13). These findings were higher than our finding among FSW, a group at a much high risk of getting HBV/HCV and STI. It appears that the national estimates of these infections among the general population could have been overestimated. Overall, the different studies conducted in Ethiopia on HBV and HCV have produced varying seroprevalence estimates. The studies have been conducted in different population groups carrying varying risks, utilized different sample sizes, and using different laboratory screening methods, some with, and others without laboratory confirmatory testing to

arrive at seroprevalence estimates. In addition, the studies were conducted in different geographic settings. These pose a major challenge in reaching a consensus on the prevalence of hepatitis viruses in Ethiopia. In light of these limitations, a large-scale seroprevalence and epidemiological studies need to be done to ensure a more robust and current national seroprevalence estimate.

The main strength of our study was the inclusion of participants from high-risk groups and being the first report on the prevalence of viral hepatitis B and C among FSW in Ethiopia at national and regional levels. The result from this study are among the first in the country and in the region, to explore both hepatitis B and C among FSW, to categorize high-risk and vulnerable population, in order to fill the gap on data of virial hepatitis in Ethiopia among key populations and in addition, this study was conducted in a large sample of FSW across 16 cities/towns of the country, which makes the findings generalizable to FSW of Ethiopia.

Nevertheless, this study also had limitations. First, as part of the national surveillance, the survey done in this round had targeted only provincial (regional) capitals, and major towns. Even in the selected cities, the presence of harder-to-reach sex worker groups like home-based sex workers might not be fully accounted for lack of detailed city maps for all regional capitals, and street names were challenges in the mapping and presentation of the results from the size estimation study. Second, as most studies were set in urban areas and as FSW were predominated included in the study, the generalizability of the results need to be considered with some caution.

Conclusion:

This study reveals an intermediate prevalence of HBV and a low prevalence of HCV infections among FSW in cities and town in Ethiopia. Our prevalence finding is lower than the estimate among the general population that was reported by previous studies. While this might have been influenced by difference in methodologies, our finding may also suggest that the coverage of

hepatitis related interventions among FSW has been effective, but require further strengthening to control HBV and HCV transmission among FSW. Scaling-up interventions for FSW such as HBV vaccination, reducing number of sexual partners, increasing condom distribution and regularly monitoring and screening for these infections and other STI among FSW is needed. FSW who are HBV and HCV infected should be informed about the transmission routes and methods to prevent further spread of the viruses. FSW testing HBV negative but not yet got vaccinated need to receive the vaccine since they were at high-risk of contracting the infection. Further epidemiological studies to determine the prevalence and determinants of HBV, HCV and other STI among different population groups are suggested.

ACKNOWLEDGMENT

The authors would like to acknowledge the Ethiopian Public Health Institute for providing materials support during this project implementation. We also thank Ethiopian Public Health institute, National HIV/AIDS surveillance and laboratory treatment center staff members for their cooperation during data collection process. We would also like to acknowledge all the Female sex workers and their partners whose data were used in this study, and all the healthcare workers who took part in the educating and treating of the included Female sex workers.

AUTHORS CONTRIBUTION

Conceptualization: Birra Bejiga Bedassa

Data curation: Birra Bejiga Bedassa, Gemechu Gudeta Ebo,Feyiso Bati Wariso, Jemal Ayalew, Saro Abdella, Sileshi Lulseged

Formal analysis: Birra Bejiga Bedassa, Gemechu Gudeta Ebo, Jemal Ayalew.

Funding acquisition: Saro Abdella, Getachew Tollera, Tsigereda Kifle

Methodology: Birra Bejiga Bedassa.

Project administration: Jaleta Bulti Tura. Getachew Tollera, Tsigereda Kifle, Saro Abdella

Resources: Jaleta Bulti Tura, Saro Abdella

Software: Jemal Ayalew**,**Gemechu Gudeta Ebo,Feyiso Bati Wariso

Supervision: Birra Bejiga Bedassa, Gemechu Gudeta Ebo, Jaleta Bulti Tura

Validation: Birra Bejiga Bedassa, Sileshi Lulseged

Visualization: Birra Bejiga Bedassa, Sileshi Lulseged.

Writing – original draft: Birra Bejiga Bedassa, Gemechu Gudeta Ebo,Feyiso Bati Wariso

Writing – review & editing: Gemechu Gudeta Ebo, Jemal Ayalew, Saro Abdella, Sileshi Lulseged

REFRENCES.

1. Ayele A, Abera D, Hailu M, Birhanu M, Desta K. Prevalence and associated risk factors for Hepatitis B and C viruses among refugees in Gambella, Ethiopia. BMC public health. 2020;20(1):1-10.
2. organization/ WWH. Global hepatitis report. Factsheet. 2017.
3. Cooke G, Lemoine M, Thursz M, Gore C, Swan T, Kamarulzaman A, et al. Viral hepatitis and the Global Burden of Disease: a need to regroup. Journal of viral hepatitis. 2013;20(9):600-1.
4. Organization/WHO/ WH. Global hepatitis report. Factsheet. 2017(19 April 2017):83.
5. Organization WH. 2016 Annual Report Communicable Diseases Cluster. 2017.
6. Giami A, Le Bail J. HIV infection and STI in the trans population: a critical review. Revue d'epidemiologie et de sante publique. 2011;59(4):259-68.

7. Organization WH. Progress report on HIV, viral hepatitis and sexually transmitted infections 2019: accountability for the global health sector strategies, 2016–2021. World Health Organization; 2019.
8. Scorgie F, Chersich MF, Ntaganira I, Gerbase A, Lule F, Lo Y-R. Socio-demographic characteristics and behavioral risk factors of female sex workers in sub-saharan Africa: a systematic review. AIDS and Behavior. 2012;16(4):920-33.
9. Puga MAM, Bandeira LM, Weis SMdS, Fernandes FRP, Castro LS, Tanaka TSO, et al. High-risk behaviors for hepatitis B and C infections among female sex workers. Revista da Sociedade Brasileira de Medicina Tropical. 2018;51:198-202.
10. Bitty-Anderson AM, Ferré V, Gbeasor-Komlanvi FA, Tchankoni MK, Sadio A, Salou M, et al. Prevalence of hepatitis B and C among female sex workers in Togo, West Africa. PloS one. 2021;16(12):e0259891.
11. Metaferia Y, Ali A, Eshetu S, Gebretsadik D. Seroprevalence and associated factors of human immunodeficiency virus, treponema pallidum, hepatitis B virus, and hepatitis C virus among female sex workers in Dessie City, Northeast Ethiopia. BioMed Research International. 2021;2021.
12. Csa I. Central statistical agency (CSA)[Ethiopia] and ICF. Ethiopia demographic and health survey, Addis Ababa, Ethiopia and Calverton, Maryland, USA. 2016.
13. Federal Ministry of Health (FMO) Addis Ababa E. National strategy for prevention and control of viral hepatitis. 2016.
14. Daka D, Hailemeskel G, Fenta DA. Seroprevalence of Hepatitis B Virus and Associated Factors Among Female Sex Workers Using Respondent-Driven Sampling in Hawassa City, Ethiopia. Infection and Drug Resistance. 2021;14:4301.

15. Moges F, Kebede Y, Kassu A, Mulu A, Tirunch M, Degu G, et al. Seroprevalence of HIV, hepatitis B infections and syphilis among street dwellers in Gondar city, Northwest Ethiopia. Ethiopian Journal of Health Development. 2006;20(3).

16. Bugssa G, Dessalegn B, Dimtsu B, Berhane Y. Prevalence and factors associated with HIV and hepatitis B virus infections among female commercial sex workers in Mekelle, Ethiopia: cross sectional study. International Journal of Pharmaceutical Sciences and Research. 2015;6(1):135.

17. Ouedraogo HG, Kouanda S, Goodman S, Lanou HB, Ky-Zerbo O, Samadoulougou BC, et al. Hepatitis B, C and delta viruses' infections and correlate factors among female sex workers in Burkina Faso, West-Africa. The Open Virology Journal. 2019;13(1).

18. Towns L, Ababa A. HIV/AIDS in Ethiopia. 2014.

19. Review WP. Ethiopia Population 2022 (Demographics, Maps, Graphs). 2022.

20. Unicef. Papua New Guinea High Frequency Phone Survey on COVID-19, December 2020 to January 2021. 2021.

21. (CSA) CSA. Ethiopia Demographic and Health Survey (2016 EDHS). 2016.

22. (EPHI) EPHI. Ethiopian Public Health Institute (EPHI). Ethiopia population-based HIV impact Assessment (EPHIA) 2017-2018:Final Report Addis Ababa: EPHIA: August 2020.EPHIA project: http://www.ephia.gov.et, The PHIA Project. 2017-2018.

23. Gile KJ, Handcock MS. Respondent-driven sampling: An assessment of current methodology. Sociological methodology. 2010;40(1):285-327.

24. (WHO) WHO. Hepatitis B. . Department of Communicable Diseases Surveillance and Response WHO/CDS/CSR/LYO 2002. 2002.

25. Rashti R, Sharafi H, Alavian SM, Moradi Y, Mohamadi Bolbanabad A, Moradi G. Systematic review and meta-analysis of global prevalence of HBsAg and HIV and HCV antibodies among people who inject drugs and female sex workers. Pathogens. 2020;9(6):432.
26. Liu J, Liang W, Jing W, Liu M. Countdown to 2030: eliminating hepatitis B disease, China. Bulletin of the World Health Organization. 2019;97(3):230.
27. Schweitzer A, Horn J, Mikolajczyk RT, Krause G, Ott JJ. Estimations of worldwide prevalence of chronic hepatitis B virus infection: a systematic review of data published between 1965 and 2013. The Lancet. 2015;386(10003):1546-55.
28. Organization WWH. Hepatitis B Key facts. Jul 27, 2021.
29. Thio C, Hawkins C. Hepatitis B Virus. Mandell, Douglas, and Bennett's Principles and Practice of Infectious Diseases. Philadelphia: Elsevier; 2020.
30. André F. Hepatitis B epidemiology in Asia, the middle East and Africa. Vaccine. 2000;18:S20-S2.
31. Rashti R, Alavian SM, Moradi Y, Sharafi H, Mohamadi Bolbanabad A, Roshani D, et al. Global prevalence of HCV and/or HBV coinfections among people who inject drugs and female sex workers who live with HIV/AIDS: a systematic review and meta-analysis. Archives of virology. 2020;165(9):1947-58.
32. Organization. WH. Global Hepatitis Report, 2017. Geneva: Switzerland: World Health Organization (WHO). 2017.
33. Organization WH. Global Hepatitis Report, 2021. Geneva: Switzerland: World Health Organization (WHO) 2021.

34. Juárez-Figueroa L, Uribe-Salas F, Conde-Glez C, Hernández-Avila M, Olamendi-Portugal M, Uribe-Zúñiga P, et al. Low prevalence of hepatitis B markers among Mexican female sex workers. Sexually transmitted infections. 1998;74(6):448-50.

35. Kassaian N, Ataei B, Yaran M, Babak A, Shoaei P. Hepatitis B and C among women with illegal social behavior in Isfahan, Iran: Seroprevalence and associated factors. Hepatitis monthly. 2011;11(5):368.

36. Papadogeorgaki H, Caroni C, Frangouli E, Flemetakis A, Katsambas A, Hadjivassiliou M. Prevalence of sexually transmitted infections in female sex workers in Athens, Greece–2005. European Journal of Dermatology. 2006;16(6):662-5.

37. Passos ADC, Figueiredo JFdC, Martinelli AdLC, Villanova MG, Nascimento MPd, Gaspar AMC, et al. Hepatitis B among female sex workers in Ribeirão Preto-São Paulo, Brazil. Revista Brasileira de Epidemiologia. 2007;10(4):517-24.

38. Mutagoma M, Nyirazinyoye L, Sebuhoro D, Riedel DJ, Ntaganira J. Syphilis and HIV prevalence and associated factors to their co-infection, hepatitis B and hepatitis C viruses prevalence among female sex workers in Rwanda. BMC infectious diseases. 2017;17(1):1-9.

39. Forbi J, Onyemauwa N, Gyar S, Oyeleye A, Entonu P, Agwale S. High prevalence of hepatitis B virus among female sex workers in Nigeria. Revista do Instituto de Medicina Tropical de São Paulo. 2008;50:219-21.

40. Kerubo G, Khamadi S, Okoth V, Madise N, Ezeh A, Abdalla Z, et al. Hepatitis B, hepatitis C and HIV-1 coinfection in two informal urban settlements in Nairobi, Kenya. PloS one. 2015;10(6):e0129247.

41. Ampofo W, Nii-Trebi N, Ansah J, Abe K, Naito H, Aidoo S, et al. Prevalence of blood-borne infectious diseases in blood donors in Ghana. Journal of clinical microbiology. 2002;40(9):3523-5.

42. Vázquez-Martínez JL, Coreño-Juárez MO, Montaño-Estrada LF, Attlan M, Gómez-Dantés H. Seroprevalence of hepatitis B in pregnant women in Mexico. salud pública de méxico. 2003;45:165-70.

43. World Health Organization UN. Joint United Nations Programme on HIV/AIDS, Global Network of Sex Work Projects. Prevention and treatment of HIV and other sexually transmitted infections for sex workers in lowand middle-income countries: recommendations for a public health approach. Geneva: World Health Organization; 2012 World Health Organization; 2012 accessed 27 July 2014.

44. Daka D, Hailemeskel G. Sero-prevalence of Hepatitis B Surface Antigen and Associated Factors Among Female Sex Workers in Hawassa, Ethiopia, 2019. 2020.

45. Desai Praseeda S, Anuradha D. A Study on the HBV and the HCV Infections in Female Sex Workers and their Co-Infection with HIV. Journal of clinical and diagnostic research: JCDR. 2013;7(2):234.

46. Magalhães RdLB, Carvalho VM, Brito GMI, de Oliveira LB, Galvão MTG, Gir E. Risk practices and immunization against hepatitis B among female sex workers. Revista da Rede de Enfermagem do Nordeste. 2016;17(5):636-42.

47. Farghaly AG, Alkassabany YM, El-Ghitany EM. HBV, HCV and HIV among female sex workers; is it a health problem? Sexual and Relationship Therapy. 2020;35(4):462-77.

48. Ishi K, Suzuku F, Saito A, Yoshimoto S, Kubota T. Prevalence of human immunodeficiency virus, hepatitis B and hepatitis C virus antibodies and hepatitis B antigen among commercial sex workers in Japan. Infectious Diseases in Obstetrics and Gynecology. 2001;9(4):215-9.
49. Todd CS, Nasir A, Stanekzai MR, Bautista CT, Botros BA, Scott PT, et al. HIV, hepatitis B, and hepatitis C prevalence and associated risk behaviors among female sex workers in three Afghan cities. AIDS (London, England). 2010;24(0 2):S69.
50. Tessema B, Yismaw G, Kassu A, Amsalu A, Mulu A, Emmrich F, et al. Seroprevalence of HIV, HBV, HCV and syphilis infections among blood donors at Gondar University Teaching Hospital, Northwest Ethiopia: declining trends over a period of five years. BMC Infectious diseases. 2010;10(1):1-7.
51. Stevens CE, Taylor PE, Pindyck J, Choo Q-L, Bradley DW, Kuo G, et al. Epidemiology of hepatitis C virus: a preliminary study in volunteer blood donors. Jama. 1990;263(1):49-53.
52. Scharara A, Christine M, Hamilton J. Hepatitis C update. Ann Intern Med. 1996;125(8):658-68.
53. Organization WH. Global progress report on HIV, viral hepatitis and sexually transmitted infections, 2021: accountability for the global health sector strategies 2016–2021: actions for impact: web annex 2: data methods. 2021.
54. Karamouzian M NM, Ghaffari Hoseini S, Mirzazadeh A. HIV and Other Sexually Transmitted Infections Among Female Sex Workers in Iran: A Systematic Review and Meta-Analysis. Arch Sex Behav. 2020;Aug;49(6):1923-37.
55. Wansbrough-Jones MH FE, Cant B, Harris K, Evans MR, Teo CG. Prevalence and genotype of hepatitis C virus infection in pregnant women and blood donors in Ghana. Trans R Soc Trop Med Hyg. 1998 Sep-Oct;92(5):496-9.

56. Koate B, Buseri F, Jeremiah Z. Seroprevalence of hepatitis C virus among blood donors in Rivers State, Nigeria. Transfusion Medicine. 2005;15(5):449-51.

57. Adjei AA, Kudzi W, Armah H, Adiku T, Amoah AB, Ansah J. Prevalence of antibodies to syphilis among blood donors in Accra, Ghana. Japanese journal of infectious diseases. 2003;56(4):165-7.

58. Matee MI, Magesa PM, Lyamuya EF. Seroprevalence of human immunodeficiency virus, hepatitis B and C viruses and syphilis infections among blood donors at the Muhimbili National Hospital in Dar Es Salaam, Tanzania. BMC public health. 2006;6(1):1-6.

59. Laurent C, Henzel D, Mulanga-Kabeya C, Maertens G, Larouze B, Delaporte E. Seroepidemiological survey of hepatitis C virus among commercial sex workers and pregnant women in Kinshasa, Democratic Republic of Congo. International journal of epidemiology. 2001;30(4):872-7.

60. Barth RE, Huijgen Q, Taljaard J, Hoepelman AI. Hepatitis B/C and HIV in sub-Saharan Africa: an association between highly prevalent infectious diseases. A systematic review and meta-analysis. International Journal of Infectious Diseases. 2010;14(12):e1024-e31.

61. Aklilu M, Messele T, Tsegaye A, Biru T, Mariam DH, Van Benthem B, et al. Factors associated with HIV-1 infection among sex workers of Addis Ababa, Ethiopia. Aids. 2001;15(1):87-96.

62. Ayele W, Nokes DJ, Abebe A, Messele T, Dejene A, Enquselassie F, et al. Higher prevalence of anti- HCV antibodies among HIV- positive compared to HIV- negative inhabitants of Addis Ababa, Ethiopia. Journal of Medical Virology. 2002;68(1):12-7.

63. Chen Y, Shen Z, Morano JP, Khoshnood K, Wu Z, Lan G, et al. Bridging the epidemic: a comprehensive analysis of prevalence and correlates of HIV, hepatitis C, and syphilis, and

infection among female sex workers in Guangxi Province, China. PloS one. 2015;10(2):e0115311.
