# Supplementary material for: Prevalence and factors associated with hepatitis B and C virus infections among female Sex workers in Ethiopia: Results of the national biobehavioral Survey, 2020
Source: PLoS One. 2022 Dec 30;17(12):e0269510. doi: 10.1371/journal.pone.0269510 (PMC9803120; doi:10.1371/journal.pone.0269510)
Supplement: Attachment — Submitted filename: Response to Reviewers.docx [file pone.0269510.s002.docx]

**Sep22, 2022**

**PONE-D-22-14713**

**Manuscript Title: Prevalence and factors associated with hepatitis B and C virus infections among female Sex workers in Ethiopia: Results of the national biobehavioral Survey, 2020**

Dear Editor,

On behalf of the authors, I would like to thank you for the review and for allowing us to revise and resubmit the manuscript to PLOS ONE. We have taken the opportunity to revise the manuscript based on the comments and suggestions from you and all three reviewers. We have also made grammatic corrections and got the manuscript reviewed by a native English speaker.

We deeply appreciate the editor’s and reviewers' comments and suggestion, which found very helpful to improve the quality of the manuscript. Per your advice, we have prepared a point-by-point response to all comments as presented below. We have also made changes as advised in a separate file titled 'Revised Manuscript with Track Changes' and have uploaded the file on per PLOS guidelines. In addition, we have uploaded three documents as part of our online submission: the tracked and clean copies of the manuscript as well as a rebuttal letter.

Sincerely

Birra Bejiga

Journal Requirements:

When submitting your revision, we need you to address these additional requirements.

1. Please ensure that your manuscript meets PLOS ONE's style requirements, including those for file naming. The PLOS ONE style templates can be found at.

**Authors’ response:** We have double-checked and ensured that all PLOS ONE’s style requirements are met.

2. We note that the grant information you provided in the ‘Funding Information’ and ‘Financial Disclosure’ sections do not match.

When you resubmit, please ensure that you provide the correct grant numbers for the awards you received for your study in the ‘Funding Information’ section.

**Authors’ response:** **We apologize for the mistake. To address this comment, we have corrected the Funding information in both online submission box and manuscript.**

3. Thank you for stating the following financial disclosure:

“This project was conducted using the effort of many institutions, organizations, and individuals without whose contributions could not have been possible like, FHAPCO, MOH, CDC, ICAP, PSI and Regional health bureau the U.S. President’s Emergency Plan for AIDS Relief (PEPFAR) funds obtained though the U.S Center for Disease Control and Prevention (CDC) under the term of cooperative agreement #U2GGH001226. The findings and conclusions in this report are those of the authors and do not necessarily represent the official position of the funding agency.”

Please state what role the funders took in the study.  If the funders had no role, please state: "The funders had no role in study design, data collection and analysis, decision to publish, or preparation of the manuscript."

If this statement is not correct you must amend it as needed.

Please include this amended Role of Funder statement in your cover letter; we will change the online submission form on your behalf.

**Authors’ response:** We have corrected the funding-related text in the manuscript and on the online submission form: “The funders had no role in study design, data collection, and analysis, decision to publish, or preparation of the manuscript.”

4. Thank you for providing a response regarding your funding information. However, it seems that it is not updated, please include again your funding information in the online submission form.

We note that the grant information you provided in the ‘Funding Information’ and ‘Financial Disclosure’ sections do not match. When you resubmit, please ensure that you provide the correct grant numbers for the awards you received for your study in the ‘Funding Information section.

**Authors’ response:** **Grant Information: The survey was conducted using funding support obtained from the Government of Ethiopia-HIV Prevention Office, Grant Number: ETH-H-HAPCO-1553.**

**Financial Disclosure: The authors do not have any financial compensation associated with the funding obtained through this grant.**

5. Thank you for stating: “No - some restrictions will apply; Due to confidentiality agreements, data can be made available subject to a non-disclosure agreement. For further information, you may contact the national data manager canter of the Ethiopian Public Health Institute at [https://ndmc.ephi.gov.et](https://ndmc.ephi.gov.et/)."

Before we proceed, please provide contact information for the data manager. Please also confirm the authors do not have special access privileges others would need when making requests.

**Author’s response: The contact information for the data manager is Mr. Lemessa Negeri the EPHI Data Manager, at the e-mail address (** [**nlemessa@gmail.com**](mailto:nlemessa@gmail.com) **). I confirm the authors do not have special access privileges.**

6. We note that you have indicated that data from this study are available upon request. PLOS only allows data to be available upon request if there are legal or ethical restrictions on sharing data publicly. For more information on unacceptable data access restrictions, please see [http://journals.plos.org/plosone/s/data-availability#loc-unacceptable-data-access-restrictions](https://nam02.safelinks.protection.outlook.com/?url=http%3A%2F%2Fjournals.plos.org%2Fplosone%2Fs%2Fdata-availability%23loc-unacceptable-data-access-restrictions&data=05%7C01%7Csl2883%40cumc.columbia.edu%7C7481eb244b724ccee07f08da796c7515%7Cb0002a9b0017404d97dc3d3bab09be81%7C1%7C0%7C637955804289544632%7CUnknown%7CTWFpbGZsb3d8eyJWIjoiMC4wLjAwMDAiLCJQIjoiV2luMzIiLCJBTiI6Ik1haWwiLCJXVCI6Mn0%3D%7C3000%7C%7C%7C&sdata=0%2FBAVW6v0jK3mz%2BeBj96GhNFHSeQSFnxinVZL6mcanQ%3D&reserved=0).

a) If there are ethical or legal restrictions on sharing a de-identified data set, please explain them in detail (e.g., data contain potentially sensitive information, data are owned by a third-party organization, etc.) and who has imposed them (e.g., an ethics committee). Please also provide contact information for a data access committee, ethics committee, or other institutional body to which data requests may be sent.

**Authors’ response:** There are no ethical and legal restrictions regarding sharing our data. In our data, there is no such information that is sensitive to any particular community. The contact information of the ethical review committee is the Scientific and Ethical Review Office (SERO) ( <https://ephi.gov.et/about-us/departments-units/scientific-and-ethical-review-office-sero/>), national data manager center (<https://ndmc.ephi.gov.et>.), Directorate of HIV and TB of Ethiopian Public Health Institute <https://ephi.gov.et/research/hiv-and-tb/>

  b) If there are no restrictions, please upload the minimal anonymized data set necessary to replicate your study findings as either Supporting Information files or to a stable, public repository and provide us with the relevant URLs, DOIs, or accession numbers. For a list of acceptable repositories, please see [http://journals.plos.org/plosone/s/data-availability#loc-recommended-repositories](https://nam02.safelinks.protection.outlook.com/?url=http%3A%2F%2Fjournals.plos.org%2Fplosone%2Fs%2Fdata-availability%23loc-recommended-repositories&data=05%7C01%7Csl2883%40cumc.columbia.edu%7C7481eb244b724ccee07f08da796c7515%7Cb0002a9b0017404d97dc3d3bab09be81%7C1%7C0%7C637955804289544632%7CUnknown%7CTWFpbGZsb3d8eyJWIjoiMC4wLjAwMDAiLCJQIjoiV2luMzIiLCJBTiI6Ik1haWwiLCJXVCI6Mn0%3D%7C3000%7C%7C%7C&sdata=HFhqoQn4h%2FsNg4%2FpSe7rxAxrXISP%2BhGnBCDncrcJCcA%3D&reserved=0).

We will update your Data Availability statement on your behalf to reflect the information you provide.

**Authors’ response:** Due to confidentiality agreements, data can be made available subject to a non-disclosure agreement. For further information, you may contact the national data manager canter of the Ethiopian Public Health Institute at https://ndmc.ephi.gov.et.

Reviewers' comments:

Reviewer's Responses to Questions

**Comments to the Author**

1. Is the manuscript technically sound, and do the data support the conclusions?

The manuscript must describe a technically sound piece of scientific research with data that supports the conclusions. Experiments must have been conducted rigorously, with appropriate controls, replication, and sample sizes. The conclusions must be drawn appropriately based on the data presented.

Reviewer #1: Yes

Reviewer #2: Yes

Reviewer #3: Yes

2. Has the statistical analysis been performed appropriately and rigorously?

Reviewer #1: No

Reviewer #2: Yes

Reviewer #3: Yes

3. Have the authors made all data underlying the findings in their manuscript fully available?

The [PLOS Data policy](https://nam02.safelinks.protection.outlook.com/?url=http%3A%2F%2Fwww.plosone.org%2Fstatic%2Fpolicies.action%23sharing&data=05%7C01%7Csl2883%40cumc.columbia.edu%7C7481eb244b724ccee07f08da796c7515%7Cb0002a9b0017404d97dc3d3bab09be81%7C1%7C0%7C637955804289544632%7CUnknown%7CTWFpbGZsb3d8eyJWIjoiMC4wLjAwMDAiLCJQIjoiV2luMzIiLCJBTiI6Ik1haWwiLCJXVCI6Mn0%3D%7C3000%7C%7C%7C&sdata=koU6r337iclzJ2UcHVhJQvlSCier7f3KnHaNe8GheCs%3D&reserved=0) requires authors to make all data underlying the findings described in their manuscript fully available without restriction, with rare exception (please refer to the Data Availability Statement in the manuscript PDF file). The data should be provided as part of the manuscript or its supporting information, or deposited to a public repository. For example, in addition to summary statistics, the data points behind means, medians and variance measures should be available. If there are restrictions on publicly sharing data—e.g. participant privacy or use of data from a third party—those must be specified.

Reviewer #1: Yes

Reviewer #2: Yes

Reviewer #3: Yes

4. Is the manuscript presented in an intelligible fashion and written in standard English?

PLOS ONE does not copyedit accepted manuscripts, so the language in submitted articles must be clear, correct, and unambiguous. Any typographical or grammatical errors should be corrected at revision, so please note any specific errors here.

Reviewer #1: No

Reviewer #2: Yes

Reviewer #3: Yes

5. Review Comments to the Author

Please use the space provided to explain your answers to the questions above. You may also include additional comments for the author, including concerns about dual publication, research ethics, or publication ethics. (Please upload your review as an attachment if it exceeds 20,000 characters)

Reviewer #1: The title of the "Prevalence and factors associated with hepatitis B and C virus infections among female Sex workers in Ethiopia: Results of the national biobehavioral Survey, 2020" topic is interesting.

**Authors’ response:** Thanks. Noted.

The sample size is big enough to provide reliable results.

**Authors’ response:** Thanks. Noted.

However, authors must clarify the highlighted comments in the attached document.

**Authors’ response:** All comments highlighted in the manuscript are addressed: Lines

The literature review is poor in the introduction section.

**Authors’ response:** The introduction section is revised addressing comments given by the reviewers. Lines 63-70 revised per comment; Line 74 acronym expanded (first entry); specified general population and FSW as commented; and refs added as suggested and required.

There is inconsistency in the method section.

The information of Ethiopia background should be summarized and focus on settings where the study has been conducted.

**Authors’ response:** Paragraph (lines 112-123) revised as suggested.

How towns and cities were selected?

**Authors’ response:** The study sites were selected purposively, and this is now described (lines 118-131).

There are no FSW in rural settings?

**.Authors’ response:** Female sex work is much less in rural areas and is not overt. Cities and major towns are the hotspots with high number of FSWs in Ethiopia and the survey focused on these areas.

It is not national representative study because some areas were excluded

**.Authors’ response:** Correct. Exclusion criteria is now included (line 139-141), and edits have been made it is now clearly spelt out that the sampling was purposive. Language has been toned down as appropriate to reflect this, including in the inferences made from the findings.

- Authors should specify the name of tests used in the study.
- Test algorithm
- Sample storage and transportation.
- Clinical management once the test is positive
- Syphilis and HIV results are not part of the study.

**Authors’ response:** These are now addressed in detail under three subtopics: Sample collection and processing and Linkage to care and treatment and Biological analysis (lines 191-228). Syphilis and HIV infection were considered as co-morbidities and included among explanatory factors. The outcome in this survey is HBV and HCV infection. Removed syphilis from among the key words.

In the abstract section authors mentioned that <=0.05 p-value was considered. 0.2 p-value is too high, it can be considered in some few key variables

**Authors’ response:** As indicated, a *P* value ≤ 0.05 was used as a cut-off to determine statistical significance for point estimates, both in the bivariable and multivariable analysis. Following the general rule, all variables achieving a *P* value of <0.2 in the bivariate analysis were included in the multiple logistic regression model (lines 40-41 and 239-242).

No incentive was provided to participants?

**Authors’ response:** Participants were compensated for their time and reimbursed the transport costs.

Median=25 and IQR=8?

**Authors’ response: Corrected. Edited.**

1. How a new participant coming without a coupon could be enrolled?
   **Authors’ response:** Each new participant was given coupons and asked to recruit three additional acquaintances. This process continued until the desired sample size was achieved and any participants without Coupon were not included in the study
2. How many waves have you registered? **Authors’ response:** The majority of the study participants were recruited from seeds that generated four to six waves. A maximum of 16 waves were attained**.**
3. Check please
   1. Consistency in data analysis:
   - Age group categories with p-values <0.05 not appearing in multivariable model. The same for number of sexual partners

**Authors’ response:** Age group categories with p-values <0.05 is now included and number of sexual partners was already in the multivariable regression model. In our analysis we have two dependent variables, so that some variables may not be candidate for one dependent variable because of their bivariate p-value > 0.2 (the modest p-value to be candidate for multivariate). That is why age at first sex being black for HBV since the bivariate p-value is greater than 0.2 and number of sexual partners for HCV and the like.

2. Multivariable model without bivariate model. What happened? Age of first sex

**Authors’ response:** This is now corrected and well in order.

3. Age at first sex selling not completed

**Authors’ response:** This is now complete.

Table 2 Should be formatted for clarity to the reader

**Authors’ response:** Formatted as suggested

The discussion section is not structured.
The author is reporting the results
The reader will be bored with this so long and light discussion

**Authors’ response:** Point well taken. Revised the discussion section addressing the comments given here and by others.

Reviewer #2: The manuscript by Bedassa et al. is well written and provides important information on the prevalence of HBV and HCV infection in Ethiopian FSWs. Although the main objective of the study was HBV and HCV, the authors provide important information about the prevalence of HIV and syphilis in this population. In my opinion, the manuscript should be published after minor modifications.
**Authors’ response:** Thanks. Noted.
1. Authors need to add study exclusion criteria. Only the inclusion criteria were placed.

**Authors’ response:** Added.

2. Which kit manufacturers are used?

**Authors’ response:** kit manufacturers**,** Sample collection and processing are now given in detail (lines 192-228).

3. It would be interesting to include the index of FSWs who did not accept to participate in the study.

**Authors’ response**: Yes, it is. Unfortunately, we do not have the data on this.

4. An important detail is the higher prevalence of HBV and anti-HCV in FSWs who reported using codons in their sexual intercourse. What could justify this finding?

**Authors’ response:** This is reviewed we have identified that it was not reported correctly. This in now corrected and it all makes sense.

5. The prevalence of HBV and anti-HCV in the study was much lower than that observed in the general population of Ethiopia. The authors suggest that this can be influenced by different methodologies. Wouldn't it be interesting to carry out an enzyme immunoassay to confirm the results (if there were financial resources for this)? Could they have too many false negatives? This is a very interesting result of the study. Another important point would be to verify the vaccination coverage for hepatitis B in this population.

**Authors’ response:** Performing enzyme immunoassay to confirm the results was very helpful, but we were unable to do this for budgetary reasons. It will be critically important for future studies in the country to include the assay. This is now indicated in the manuscript (lines 434-436). In Ethiopia’s HBV vaccination program only the health workers among adult population are eligible for the HBV vaccination. FSW are also not targeted by the program, and we have no data on HBV vaccination coverage among FSW in the country.

Reviewer #3: The authors did a great job giving relevance to aspects of the epidemiology of infectious agents in vulnerable populations and a very rich statistical analysis with a huge sample.
**Authors’ response:** Thank you. Noted.

Nonetheless, some excerpts can be improved to provide a better reading and understanding of the article.
1. The authors should clarify the following sections to avoid confusion - lines 254 through 259 - the excerpt should focus on co-infection with HIV-positive cases despite HIV-negative cases.
**Authors’ response:** The section (now lines 289-292) is revised and made much easier to understand.

2. Tables 1 to 4 should include a column to add the 95% CI

**Authors’ response:** 95% confidence intervals added to tables 1-4

3. Table 1 presents duplicate entries
**Authors’ response:** All corrected.

4. There are some misspelled words - line 65 "HVC"; line 115-116 duplicated "in 2016"; line 122 - "crass"; line 184 "a liquated"; line 239 "chat"; line 354 "STS".

**Authors’ response:** All corrected.

5. In the results section, under the topic Factors associated with hepatitis B and C, please provide a clearer explanation of what the COR parameter is about. Is it the same AOR presented in the data analysis topic and was it just typed wrong? if so, change the acronym COR to AOR whenever it's mistaken, if not, provide an adequate explanation of how this statistical result was obtained and its purpose.
**Authors’ response:** COR is Crude Odds Ratio, the point estimate we get from the bivariate analysis and has its associated 95%CI and *P*-value. Factors which achieve *P*-values ≤0.5 are considered statistically significant. These along with others which achieved *P*-values of <0.2 are inserted in the multivariable regression model. What is presented in the table and the description under factors associated with HBV/HCV is the results of the bivariable analysis with COR and the multivariable analysis with associated AOR (Adjusted Odds Ratio).

6. At the beginning of the discussion section, lines 355 and 356, it would be better to present the reference right after the mention of the previous study cited in this paragraph.
**Authors’ response:** The discussion section revised taking this comment and other to shorten and make the section crisper.

7. Review the score on line 321, there should be a point after endemicity.

**Authors’ response:** Done

8. The authors should revise the language to improve readability in lines 340 to 344 to make it more understandable. Do the same for the lines 357 and 358; 371 to 374; 385 to 389; 395 to 397,on line 436 where it starts with ... , a group.

**Authors’ response: The discussion section is r**evised based on these and other comments

6. PLOS authors have the option to publish the peer review history of their article ([what does this mean?](https://nam02.safelinks.protection.outlook.com/?url=https%3A%2F%2Fjournals.plos.org%2Fplosone%2Fs%2Feditorial-and-peer-review-process%23loc-peer-review-history&data=05%7C01%7Csl2883%40cumc.columbia.edu%7C7481eb244b724ccee07f08da796c7515%7Cb0002a9b0017404d97dc3d3bab09be81%7C1%7C0%7C637955804289544632%7CUnknown%7CTWFpbGZsb3d8eyJWIjoiMC4wLjAwMDAiLCJQIjoiV2luMzIiLCJBTiI6Ik1haWwiLCJXVCI6Mn0%3D%7C3000%7C%7C%7C&sdata=8fHbZSWoFschVzEEC3y7phgonnyxtBiixRqU7nW2qWk%3D&reserved=0)). If published, this will include your full peer review and any attached files.


If you choose “no”, your identity will remain anonymous but your review may still be made public.


**Do you want your identity to be public for this peer review?** For information about this choice, including consent withdrawal, please see our [Privacy Policy](https://nam02.safelinks.protection.outlook.com/?url=https%3A%2F%2Fwww.plos.org%2Fprivacy-policy&data=05%7C01%7Csl2883%40cumc.columbia.edu%7C7481eb244b724ccee07f08da796c7515%7Cb0002a9b0017404d97dc3d3bab09be81%7C1%7C0%7C637955804289544632%7CUnknown%7CTWFpbGZsb3d8eyJWIjoiMC4wLjAwMDAiLCJQIjoiV2luMzIiLCJBTiI6Ik1haWwiLCJXVCI6Mn0%3D%7C3000%7C%7C%7C&sdata=L42fznYFS49ZL4jvi995%2B6YqrN5%2FC2viudL7tqDWwC8%3D&reserved=0).

Reviewer #1: **Yes:** Mutagoma Mwumvaneza

Reviewer #2: **Yes:** Luiz Fernando Almeida Machado

Reviewer #3: **Yes:** Rogério Valois Laurentino

 [NOTE: If reviewer comments were submitted as an attachment file, they will be attached to this email and accessible via the submission site. Please log into your account, locate the manuscript record, and check for the action link "View Attachments". If this link does not appear, there are no attachment files.]

While revising your submission, please upload your figure files to the Preflight Analysis and Conversion Engine (PACE) digital diagnostic tool, [https://pacev2.apexcovantage.com/](https://nam02.safelinks.protection.outlook.com/?url=https%3A%2F%2Fpacev2.apexcovantage.com%2F&data=05%7C01%7Csl2883%40cumc.columbia.edu%7C7481eb244b724ccee07f08da796c7515%7Cb0002a9b0017404d97dc3d3bab09be81%7C1%7C0%7C637955804289544632%7CUnknown%7CTWFpbGZsb3d8eyJWIjoiMC4wLjAwMDAiLCJQIjoiV2luMzIiLCJBTiI6Ik1haWwiLCJXVCI6Mn0%3D%7C3000%7C%7C%7C&sdata=ZQC0rSH0qdlQV%2Fsj21oLmegUfCtdxbs9EVtIXjr%2F34o%3D&reserved=0). PACE helps ensure that figures meet PLOS requirements. To use PACE, you must first register as a user. Registration is free. Then, login and navigate to the UPLOAD tab, where you will find detailed instructions on how to use the tool. If you encounter any issues or have any questions when using PACE, please email PLOS at [figures@plos.org](mailto:figures@plos.org). Please note that Supporting Information files do not need this step.

*In compliance with data protection regulations, you may request that we remove your personal registration details at any time.* [*(Remove my information/details)*](https://nam02.safelinks.protection.outlook.com/?url=https%3A%2F%2Fwww.editorialmanager.com%2Fpone%2Flogin.asp%3Fa%3Dr&data=05%7C01%7Csl2883%40cumc.columbia.edu%7C7481eb244b724ccee07f08da796c7515%7Cb0002a9b0017404d97dc3d3bab09be81%7C1%7C0%7C637955804289544632%7CUnknown%7CTWFpbGZsb3d8eyJWIjoiMC4wLjAwMDAiLCJQIjoiV2luMzIiLCJBTiI6Ik1haWwiLCJXVCI6Mn0%3D%7C3000%7C%7C%7C&sdata=LX%2BGKs%2FTmHVqNFTnqt5onSCZXMG076NHv23OdDo5M2E%3D&reserved=0)*. Please contact the publication office if you have any questions.*

--

*With Best Regards*

*Birra Bejiga Bedassa (MPH, Epidemiology and Biostatistics)*

*Ethiopian Public Health Institute TB/HIV Directorate; HIV Surveillance Team*

*Email:* [*birr4allephi@gmail.com/*](https://nam02.safelinks.protection.outlook.com/?url=http%3A%2F%2Fbirr4allephi%40gmail.com%2F&data=05%7C01%7Csl2883%40cumc.columbia.edu%7C7481eb244b724ccee07f08da796c7515%7Cb0002a9b0017404d97dc3d3bab09be81%7C1%7C0%7C637955804289544632%7CUnknown%7CTWFpbGZsb3d8eyJWIjoiMC4wLjAwMDAiLCJQIjoiV2luMzIiLCJBTiI6Ik1haWwiLCJXVCI6Mn0%3D%7C3000%7C%7C%7C&sdata=Q9RBoNu6jx4STiSLmGQ3V8xIYDyyLy%2BIFZodxQ1w0uU%3D&reserved=0) [*birr4all@gmail.com*](mailto:birr4all@gmail.com)

*ORcid:*[*https://orcid.org/my-orcid?orcid=0000-0002-2784-3564*](https://nam02.safelinks.protection.outlook.com/?url=https%3A%2F%2Forcid.org%2Fmy-orcid%3Forcid%3D0000-0002-2784-3564&data=05%7C01%7Csl2883%40cumc.columbia.edu%7C7481eb244b724ccee07f08da796c7515%7Cb0002a9b0017404d97dc3d3bab09be81%7C1%7C0%7C637955804289544632%7CUnknown%7CTWFpbGZsb3d8eyJWIjoiMC4wLjAwMDAiLCJQIjoiV2luMzIiLCJBTiI6Ik1haWwiLCJXVCI6Mn0%3D%7C3000%7C%7C%7C&sdata=OzOA%2F%2Fw1FSjkJSBcJTGWD4rmFwfEu2kV%2BnMjrxr8RDE%3D&reserved=0)

*contact Number:  +251 913687219*
