# Supplementary material for: Prevalence and factors associated with hepatitis B and C virus infections among female Sex workers in Ethiopia: Results of the national biobehavioral Survey, 2020
Source: PLoS One. 2022 Dec 30;17(12):e0269510. doi: 10.1371/journal.pone.0269510 (PMC9803120; doi:10.1371/journal.pone.0269510)
Supplement: Attachment — Submitted filename: Response to Reviewers.docx [file pone.0269510.s003.docx]

**NOV 21, 2022**

**PONE-D-22-14713**

**Manuscript Title: Prevalence and factors associated with hepatitis B and C virus infections among female Sex workers in Ethiopia: Results of the national biobehavioral Survey, 2020**

Dear Editor,

On behalf of myself and the authors, I would like to thank you for a and the reviewers for having critically reviewed the manuscript and for giving allowing us to revise and re-resubmit it to PLOS ONE. We have revised the manuscript based on the comments and suggestions from you and all two reviewers.

We deeply appreciate yours and the reviewers' comments and suggestions, which we found very helpful and used to improve the quality of the manuscript. Per your advice, we have prepared a point-by-point response to all comments as presented below. We have also made changes as advised in a separate file titled 'Revised Manuscript with Track Changes' and have uploaded the file per PLOS guidelines. In addition, we have uploaded three documents as part of our the online submission: the tracked and clean copies of the manuscript as well as a rebuttal letter.

Sincerely

Birra Bejiga

Journal Requirements:

When submitting your revision, we need you to address these additional requirements.

Please review your reference list to ensure that it is complete and correct. If you have cited papers that have been retracted, please include the rationale for doing so in the manuscript text, or remove these references and replace them with relevant current references. Any changes to the reference list should be mentioned in the rebuttal letter that accompanies your revised manuscript. If you need to cite a retracted article, indicate the article’s retracted status in the References list and also include a citation and full reference for the retraction notice

**Authors’ response:** We have double-checked and ensured that all is complete and correct.

Reviewer's Responses to Questions

**Comments to the Author**

1. If the authors have adequately addressed your comments raised in a previous round of review and you feel that this manuscript is now acceptable for publication, you may indicate that here to bypass the “Comments to the Author” section, enter your conflict of interest statement in the “Confidential to Editor” section, and submit your "Accept" recommendation.

Reviewer #2: (No Response)

Reviewer #3: (No Response)

2. Is the manuscript technically sound, and do the data support the conclusions?

The manuscript must describe a technically sound piece of scientific research with data that supports the conclusions. Experiments must have been conducted rigorously, with appropriate controls, replication, and sample sizes. The conclusions must be drawn appropriately based on the data presented.

Reviewer #2: Yes

Reviewer #3: Yes

**Authors’ response:** Thanks. Noted.

3. Has the statistical analysis been performed appropriately and rigorously?

Reviewer #2: Yes

Reviewer #3: Yes

**Authors’ response:** Thanks. Noted.

4. Have the authors made all data underlying the findings in their manuscript fully available?

The [PLOS Data policy](http://www.plosone.org/static/policies.action#sharing) requires authors to make all data underlying the findings described in their manuscript fully available without restriction, with rare exception (please refer to the Data Availability Statement in the manuscript PDF file). The data should be provided as part of the manuscript or its supporting information, or deposited to a public repository. For example, in addition to summary statistics, the data points behind means, medians and variance measures should be available. If there are restrictions on publicly sharing data—e.g. participant privacy or use of data from a third party—those must be specified.

Reviewer #2: Yes

Reviewer #3: Yes

**Authors’ response:** Thanks. Noted.

5. Is the manuscript presented in an intelligible fashion and written in standard English?

PLOS ONE does not copyedit accepted manuscripts, so the language in submitted articles must be clear, correct, and unambiguous. Any typographical or grammatical errors should be corrected at revision, so please note any specific errors here.

Reviewer #2: Yes

Reviewer #3: Yes

**Authors’ response:** Thanks. Noted.

6. Review Comments to the Author

Please use the space provided to explain your answers to the questions above. You may also include additional comments for the author, including concerns about dual publication, research ethics, or publication ethics. (Please upload your review as an attachment if it exceeds 20,000 characters)

Reviewer #2: I would like to thank you for the opportunity to review the manuscript by Bedassa et al. and congratulate the authors for the information regarding the epidemiology of HBV and HCV in Ethiopian FSWs. The manuscript is well written but, in my opinion, needs some adjustments to be published.

**Authors’ response:** Thanks. Noted.

 Line 66: put HIV in full before the acronym

**Authors’ response:** lines 66 revised as suggested

In the introduction put the prevalence of HCV in FSWs in Ethiopia

**Authors’ response: revised and added as suggested.**

- Line 295: state that 184 were infected with HBV or had HCV Ab, because from the way it is written, you can understand that it was HBV-HCV co-infection.

**Authors’ response:** lines 295 revised as suggested.

My main suggestion for authors to add the prevalence of HBV and HCV Ab in each city (could be in a table) to demonstrate the location(s) of highest prevalence, which could help in decision making regarding the fight against infection by the health authorities.

**Authors’ response: revised and added as suggested.**

Reviewer #3: There are several typing corrections that should be looked at more carefully considering maintaining a standard in writing the article, this attention must be given to the spacing of the text for the references that are in parentheses, for example, all citations should follow the same pattern presented in line 63, being clearer and more specific, several times the space was not given in typing between the text and parenthesis of the reference, this needs to be corrected in lines 65, 70, 72, 74, 88, 89, 90, 93, 95, 98, 103, 105, 222, 339, 341, 342, 343, 344, 346, 382, 385, 387, 394, 399, 402, 413. In all these lines, the typing should be like this: are endemic in developing countries (1) [note the space before the quote] instead of like this: .. ..mostly from hepatitis-related cirrhosis and liver cancer(2) [without the space before the citation].
 **Authors’ response: thank you Revised as suggested.**

In line 103, a space must be inserted after the period in the excerpt: ...(12).FSW have been identified as a population group with the highest risk for STI...
 **Authors’ response: thank you Revised as suggested.**

In lines 106/107 it should be written: to determine instead of to determined in the excerpt: there is no national data among FSW to determined HBV and HCV prevalence and driving factors

**Authors’ response: thank you for your observation Revised as suggested.**

In line 114 the word "in" after the period must be capitalized in the passage: ... 120 million and had low per capita income (22). in 2016, when the...
 **Authors’ response: thank you Revised as suggested.**

In line 188 it should be written "aliquoted" instead of "a liquidated" in the passage: the plasma was separated and a liquidated in two...
 **Authors’ response: thank you Revised**

In line 192, a space must be inserted before the abbreviation EPHI in the passage: teams from Ethiopian Public Health institute(EPHI).

**Authors’ response: thank you Revised**

In table 1, the font used in the 95% confidence interval column is clearly one size larger than the rest of the table, standardizing for the same font and font size

**Authors’ response: thank you Revised as suggested.**

In line 363 the dot must be after the second parenthesis in the passage: countries.(18, 36-41)

**Authors’ response: thank you Revised as suggested.**

In line 373 it should be "scarce" instead of "scare"

**Authors’ response: thank you Revised as suggested.**

In line 388 it should be "might be related to" instead of "might related to"
 **Authors’ response: thank you Revised**
In line 400 it should be "through" instead of "though"

**Authors’ response: thank you Revised as suggested.**

Line 401 should not have the dot in the section: ...injection equipment (56).or rather commonly...

**Authors’ response: thank you Revised**

In line 443 it should be: "These factors need to be taken" instead of "These need factors to be taken into"

**Authors’ response: thank you Revised**

7. PLOS authors have the option to publish the peer review history of their article ([what does this mean?](https://journals.plos.org/plosone/s/editorial-and-peer-review-process#loc-peer-review-history)). If published, this will include your full peer review and any attached files.


If you choose “no”, your identity will remain anonymous but your review may still be made public.


**Do you want your identity to be public for this peer review?** For information about this choice, including consent withdrawal, please see our [Privacy Policy](https://www.plos.org/privacy-policy).

Reviewer #2: No

Reviewer #3: **Yes:** Rogério Valois Laurentino

[NOTE: If reviewer comments were submitted as an attachment file, they will be attached to this email and accessible via the submission site. Please log into your account, locate the manuscript record, and check for the action link "View Attachments". If this link does not appear, there are no attachment files.]

While revising your submission, please upload your figure files to the Preflight Analysis and Conversion Engine (PACE) digital diagnostic tool, <https://pacev2.apexcovantage.com/>. PACE helps ensure that figures meet PLOS requirements. To use PACE, you must first register as a user. Registration is free. Then, login and navigate to the UPLOAD tab, where you will find detailed instructions on how to use the tool. If you encounter any issues or have any questions when using PACE, please email PLOS at [figures@plos.org](mailto:figures@plos.org). Please note that Supporting Information files do not need this step.

*In compliance with data protection regulations, you may request that we remove your personal registration details at any time.* [*(Remove my information/details)*](https://www.editorialmanager.com/pone/login.asp?a=r)*. Please contact the publication office if you have any questions.*--

*With Best Regards*

*Birra Bejiga Bedassa (MPH, Epidemiology and Biostatistics)*

*Ethiopian Public Health Institute TB/HIV Directorate; HIV Surveillance Team*

*Email:* [*birr4allephi@gmail.com/*](https://nam02.safelinks.protection.outlook.com/?url=http%3A%2F%2Fbirr4allephi%40gmail.com%2F&data=05%7C01%7Csl2883%40cumc.columbia.edu%7C7481eb244b724ccee07f08da796c7515%7Cb0002a9b0017404d97dc3d3bab09be81%7C1%7C0%7C637955804289544632%7CUnknown%7CTWFpbGZsb3d8eyJWIjoiMC4wLjAwMDAiLCJQIjoiV2luMzIiLCJBTiI6Ik1haWwiLCJXVCI6Mn0%3D%7C3000%7C%7C%7C&sdata=Q9RBoNu6jx4STiSLmGQ3V8xIYDyyLy%2BIFZodxQ1w0uU%3D&reserved=0) [*birr4all@gmail.com*](mailto:birr4all@gmail.com)

*ORcid:*[*https://orcid.org/my-orcid?orcid=0000-0002-2784-3564*](https://nam02.safelinks.protection.outlook.com/?url=https%3A%2F%2Forcid.org%2Fmy-orcid%3Forcid%3D0000-0002-2784-3564&data=05%7C01%7Csl2883%40cumc.columbia.edu%7C7481eb244b724ccee07f08da796c7515%7Cb0002a9b0017404d97dc3d3bab09be81%7C1%7C0%7C637955804289544632%7CUnknown%7CTWFpbGZsb3d8eyJWIjoiMC4wLjAwMDAiLCJQIjoiV2luMzIiLCJBTiI6Ik1haWwiLCJXVCI6Mn0%3D%7C3000%7C%7C%7C&sdata=OzOA%2F%2Fw1FSjkJSBcJTGWD4rmFwfEu2kV%2BnMjrxr8RDE%3D&reserved=0)

*contact Number:  +251 913687219*
